# The Experimentalist’s Guide to Machine Learning for Small Molecule Design

**DOI:** 10.1021/acsabm.3c00054

**Published:** 2023-08-03

**Authors:** Sarah E. Lindley, Yiyang Lu, Diwakar Shukla

**Affiliations:** † Department of Bioengineering, University of Illinois, Urbana−Champaign, Illinois 61801, United States; ‡ Department of Chemical and Biomolecular Engineering, University of Illinois, Urbana−Champaign, Illinois 61801, United States; § Center for Biophysics & Computational Biology, University of Illinois, Urbana−Champaign, Illinois 61801, United States; ⊥ Department of Plant Biology, University of Illinois, Urbana−Champaign, Illinois 61801, United States

**Keywords:** small molecule design, drug design, machine learning, data analysis, QSAR, experimentalist friendly

## Abstract

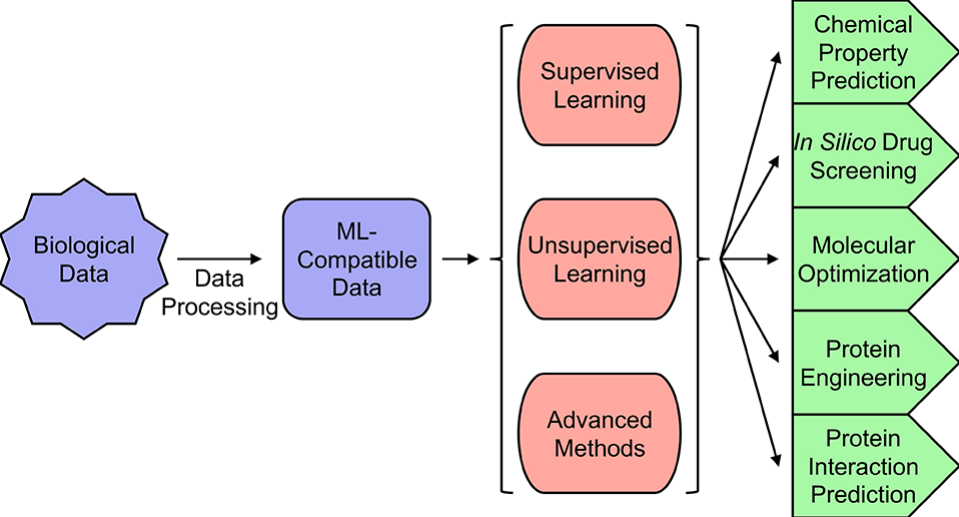

Initially part of the field of artificial intelligence, machine learning (ML) has become a booming research area since branching out into its own field in the 1990s. After three decades of refinement, ML algorithms have accelerated scientific developments across a variety of research topics. The field of small molecule design is no exception, and an increasing number of researchers are applying ML techniques in their pursuit of discovering, generating, and optimizing small molecule compounds. The goal of this review is to provide simple, yet descriptive, explanations of some of the most commonly utilized ML algorithms in the field of small molecule design along with those that are highly applicable to an experimentally focused audience. The algorithms discussed here span across three ML paradigms: supervised learning, unsupervised learning, and ensemble methods. Examples from the published literature will be provided for each algorithm. Some common pitfalls of applying ML to biological and chemical data sets will also be explained, alongside a brief summary of a few more advanced paradigms, including reinforcement learning and semi-supervised learning.

## Introduction

**Figure 1:**
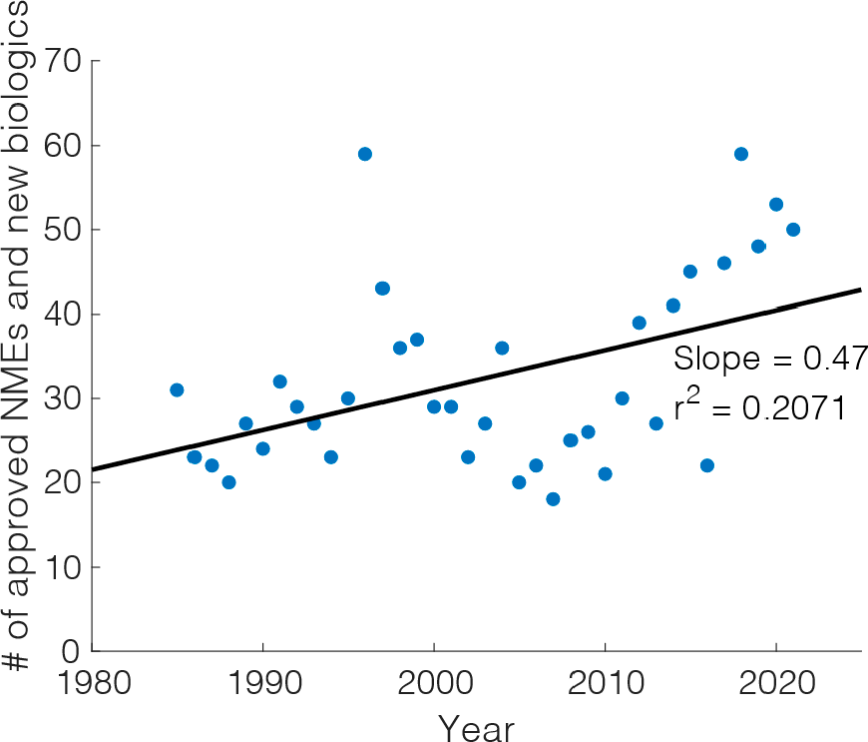
Amount of new molecular entities (NMEs) and new biologics approved by US Food and Drug Administration each year, 1980–2021. Blue dots represent raw data, and the black line represents the best linear fit to the data. The resulting linear fit shows a gradual increase in number of approved NMEs and new biologicals every year, but with low statistical significance (*r*^2^ = 0.2071). Data obtained from US Food and Drug Administration.^15^

Small molecule design is an arduous process that involves the identification of the initial lead compound, optimization of its chemical structure, determination of dosage and off-target effects, and validation of its efficacy.^1−3^ Each of these steps is riddled with experimentally intensive protocols. For the initial identification, up to tens of thousands of molecular candidates are required to be experimentally tested against a biological model.^4−7^ The model used in this step needs to be easily scalable while still maintaining high accuracy to the molecule’s target system. During optimization, many compounds that are structurally^8,9^ or mechanistically^10,11^ similar to the lead compound are screened on a model system to identify improved candidates. Dose response experiments are necessary for pinpointing the optimal dosage, which increases the amount of experimental efforts required. Improved experimental model systems with enhanced similarity to target settings (such as plant or animal models), as well as extensive assays on various aspects of the model, are needed for further validation of the potency of the candidates and the identification of potential off-target effects.^12−14^ Finally, multiple subsequent trials of increasing scale and scope are conducted to validate the efficacy and safety of the developed molecule. This step filters out a large portion of small molecule candidates, and only a small fraction will be approved by the relevant government agency such as the Food and Drug Administration (FDA) or the United States Department of Agriculture. For example, the number of new molecular entities (NMEs), drugs with a method of action that is novel to the FDA, and Biologics, novel therapeutics from a living source, that have been discovered and approved has remained relatively stable since the 1980s (Figure 1).^15^ This stable rate of discovery is no match for the increasing need for new therapeutics, as new medical challenges such as Ebola, SARS-CoV-2, and monkeypox continue to arise and must be accelerated through the introduction of modern techniques.

In the past, small molecule design required extensive and time-consuming experimental investigations. Recently, the continued improvements in computer hardware,^16,17^ in combination with advancements in novel computational algorithms,^18^ have provided an unprecedented opportunity to accelerate the development process. Such levels of computing power have been frequently utilized in computational biology and have been applied to a variety of research areas, ranging from protein engineering,^19−22^ drug design and optimization,^23−25^ large-scale modeling of biological systems,^26−29^ and clinical diagnostics.^30−33^ In this review, the applications of machine learning (ML) algorithms on small molecule design will be discussed.

## A Brief History of Small Molecule Design

Historically, the field of small molecule design was preceded by the field of drug development, which relied heavily on compounds within extracts from naturally occurring sources. After an extract’s activity is verified, a series of purifications are needed to narrow down the scope of potential active constituents until the active compound is identified.^34^ Such constituents, termed lead compounds, are usually difficult to purify, may be available only in small quantities with an unclear mechanism of action, and may be structurally complicated. Penicillin, one of the most famous antibiotics, was initially discovered by Alexander Fleming as a crude extract from the mold *P. rubens*. The effectiveness of the extract was demonstrated in his publication in 1929,^35^ but the purified compound was not isolated until 1940,^36^ which exemplifies the inherent difficulty of utilizing extracts from natural sources for drug development.

In the past few decades, the rapidly developing fields of chemistry and biology have given rise to methodologies to generate drug candidates without the use of biological extracts. These techniques resulted in the emergent field of small molecule design and increased the rate of compound testing in the pharmaceutical industry, while time consumption and labor costs remained relatively low. In 1992, the first combinatorically generated small molecule compound library was reported.^37^ The library was synthesized by the rapid assembly of various functional groups onto a small molecule scaffold, creating a library of derivative compounds from the given scaffold.^37^ Following that, many small molecule libraries of compounds based on scaffolds were generated and tested,^38,39^ and many research groups focused on scaffolds that showed activity toward multiple biological targets, termed privileged scaffolds, for their research.^40,41^

**Figure 2:**
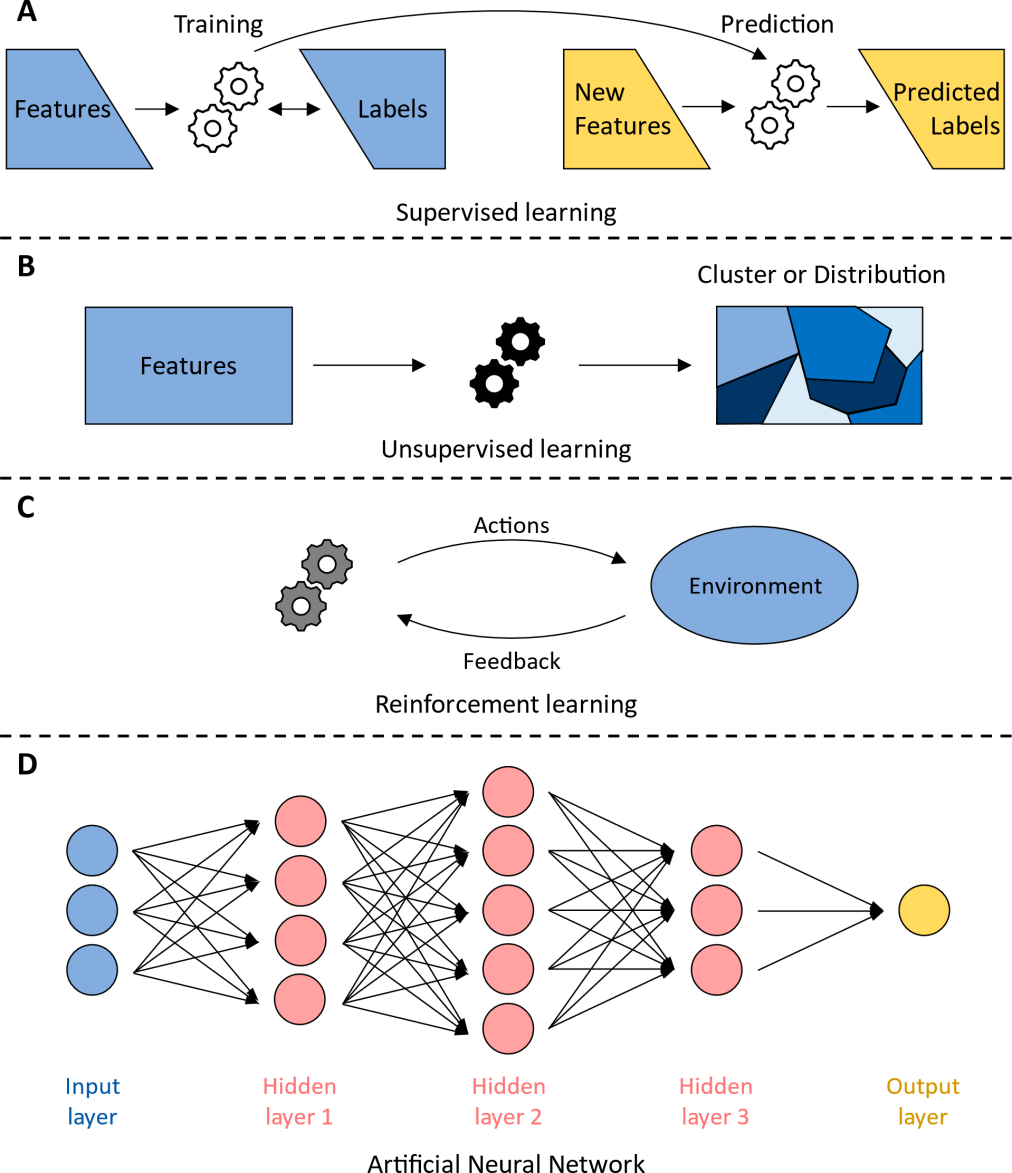
Four widely applicable types of machine learning algorithms. (A) Supervised learning attempt to learn the relationship between existing features and labels by training a model (left). After training, the model is used to predict labels for a new set of unlabeled features. (B) Unsupervised learning aims to infer useful information from features only. The output of unsupervised learning is usually in the form of grouping/clusters or distributions. (C) Reinforcement learning, instead of attempting to directly learn from existing data sets, aims to explore a well-defined environment. It takes iterative actions in the environment, and in turn the environment provides feedback about if the action is desirable or not. The algorithm then adjusts its next action according to past feedback, and the cycle continues. (D) An artificial neural network (ANN) is structured in a layered fashion. Each layer contains a number of basic calculation units called neurons (circles), which calculates a weighted sum of all the outputs from the neurons in the previous layer, and sends the sum as its own output to the neurons in the next layer. A typical ANN consists of an input layer, a number of hidden layers (3 pictured), and an output layer.

Even though the rapid synthesis of scaffold-based compound libraries is many orders of magnitude faster than isolating and purifying active components from extracts in nature, the process of small molecule design still suffers from the large amount of time and effort needed to test the synthesized compounds, which limits the rate of discovery. Recent decades have seen major improvements in ML theories and algorithms, and these methods have been increasingly and widely used in the context of molecular design. In short, the goal of ML is to develop algorithms that incorporate existing data into a suitable model to predict unobserved results. In the context of small molecule design, ML algorithms have the potential to learn from existing chemical and biological data sets to predict the activities of untested compounds. ML originated in the field of artificial intelligence before branching off and flourishing in the 1990s.^42^ Since then, a great many algorithms have emerged from the enormous efforts of researchers worldwide. These algorithms range from the simple linear regression, where a linear functional fit is constructed for a given data set,^43,44^ to the highly complicated and nonlinear deep neural network, which contains tens to hundreds of basic calculation units called neurons that communicate among each other to predict an output.^44,45^ These ML algorithms can a) learn from the structure and effects of compounds reported in literature and generate new candidates for testing, b) utilize previously trained models from past research to adapt to a new problem with a small amount of available data, and c) predict the effect of molecules from an untested library to allow for prioritization during the testing procedure.^46−49^ There are four widely applicable approaches to ML: supervised learning, unsupervised learning, reinforcement learning, and artificial neural networks. Supervised learning requires data sets containing both features (or independent variables) and labels (or dependent variables). It aims to develop a model that best fits the relationship of the features and labels, and then use that model to predict labels from new features (Figure 2A). These methods are well-suited for molecular design tasks where abundant data with high quality annotations exist and can provide reliable guidelines and suggestions to potential hit compounds. Unsupervised learning only requires labels to function, and it aims to uncover a clustering (or grouping) or a distribution of the features (Figure 2B). These methods are most applicable to situations where large-scale data sets exist for the molecular design target, but few to no annotations can be found. They will provide a quick and easy way of categorizing unlabeled data. Reinforcement learning tackles tasks that require exploration of a well-defined environment by iteratively carrying out actions and receiving feedback from the environment in the form of increases or decreases in score. The algorithm then adjusts its next action according to the score it receives. For example, if the goal is to design an inhibitor based on a chemical scaffold, then modifications of the scaffold that result in inhibition will result in an increased score, with similar modifications being more likely to be repeated. On the other hand, modifications that result in activation will result in a decreased score, and similar modifications are less likely to be repeated (Figure 2C). Reinforcement learning is uniquely suitable for design tasks where large-scale data sets are unavailable, but a general chemical space for exploration can be deduced from past research. It can provide a stepwise approach to designing molecules with desired properties. Finally, the artificial neural network mimics the structure of biological neurons to extract information from input data. It consists of multiple layers of basic calculation units called neurons, which calculate a weighted sum of all the outputs from the neurons in the previous layer and send the sum as its own output to the neurons in the next layer. A typical ANN consists of an input layer, several hidden layers (3 pictured in Figure 2D), and an output layer (Figure 2D). ANN provides a generalizable structure for many machine learning tasks, but its strength lies in its ability to handle extremely large data sets. Thus, ANN is highly suitable for molecular design tasks where data sets from large scale high-throughput experiments are involved. In the following sections, simple, yet commonly utilized, ML algorithms will be introduced, and their advantages and weaknesses will be discussed. Three categories of ML algorithms will be covered: supervised learning, unsupervised learning, and advanced methods. The algorithms addressed within these categories were selected due to their applicability to small molecule discovery.

## Data Processing

Prior to delving into various ML models and approaches, one must understand the types of data available as well as the types that are necessary for each ML model to function properly. This section will provide background information and terminology to provide an adequate understanding of key concepts before proceeding further.

### Types of Data

Perhaps the most basic concept to define is the independent vs the dependent variable. The independent variable is the factor that you, as the researcher, manipulate to determine their effect on the dependent variable. In ML contexts, these are frequently referred to as the features of your data set. The dependent variable is the output, often collected from experimental results, termed "labels" in ML contexts. The data for these two types of variables can take many forms depending on the subject studied and the needs and structure of the experiment. These data can be broken down into quantitative and qualitative data. Quantitative data are data that can be expressed in some numerical form, which can typically be broken down into two categories: continuous and discrete. Continuous data can take the form of any number or portion of a number. These data often represent things that are measurable to a high level of precision such as concentration, length, time, volume, etc. In contrast, discrete data must be in the form of whole numbers or integers and are often seen as things that can be counted such as the number of individuals, items, molecules, functional groups, etc. Qualitative data, on the other hand, deal with things that can only be roughly measured and frequently expressed with categorical labels. This data type can be further divided into two categories: nominal and ordinal. Nominal data deal with information such as gender, religion, nationality, etc. They can be represented as numbers by assigning a unique integer to each of the types (such as types of beverages), but the numbers are not understood as an indication of relative quality or ranking. In contrast, ordinal data deal with information that has an order as an integral part of its identity; examples can be seen in grades, military ranks, or satisfaction ratings. Ordinal data can take a numerical form, but the numbers are only in relation to the ranking between the different values of the same feature and cannot be interpreted in comparison to values of a different feature. For example, if numbers are assigned to grades from the lowest to the highest (F - 1, D - 2, C - 3, B - 4, A - 5), then a grade of 4 is better than a grade of 2. However, a grade of 4 cannot be compared to a satisfaction rating of 4, because the two numbers are not derived from the same set of ordinal data.

### Data Cleaning and Wrangling

Large chemical and biological data sets obtained from experiments commonly suffer from mistakes due to both instrumental and human errors. Some common forms of errors include missing values, duplicate entries, and outliers. It is crucial to examine and curate your input data set before feeding it into your ML algorithm of choice because any error in it will be picked up and learned by the algorithm, which will most likely cause a significant derailment in the performance of the algorithm.^50^ Though it would be extremely difficult and time-consuming to identify every error manually, it would be comparatively easy and expedient to write a simple program to iterate through the whole data set and check for potential problems, prompting for human input only if necessary. Missing values can be easy to identify but can be tricky to deal with depending on their prevalence in your data set. The simplest method to correct for missing values is to delete the variables or observations with them. However, when missing values are highly prevalent in the entire data set, deleting variables or observations may end up removing most of the data. An alternative method is to substitute missing values with estimations. The simplest estimation is the average of all existing values of the same variable.^50^ If there are other correlated variables in the data set, linear regressions can be utilized to provide an estimate of the missing values as well.^50^ Duplicate entries are generally easy to resolve by simply removing them but can prove challenging to identify. While exact duplicates do exist, in biological and chemical data sets, they manifest more frequently as extremely similar entries or entries with duplicated values in a select few variables.^50^ Highly similar entries are usually the result of unintentionally duplicated experiments. However, due to the inherent randomness of chemical and biological processes, distinct experiments can also yield similar results. Thus, when correcting these entries, it is crucial to ensure that the experiments producing these entries are actual duplicates. This can be done by checking various aspects of the entries, including the chemical identifiers, experimental conditions, cell line identifiers, etc. Entries with duplicated values in some but not all variables require extra caution. Each duplicated value needs to be manually evaluated with expert knowledge to determine to which entry the value truly belongs to. For the other entries with duplicated values, they should be assumed to be missing in these values and amended accordingly using the methods mentioned above for missing values. In the case where manual evaluation cannot determine to which entry the duplicated value belongs, it may be beneficial to simply remove all entries affected by the duplication. Finally, outlier entries can generally be picked out through data visualization. One common method is to create a scatter plot of the variables that you wish to examine. Then outlier values can be picked out through a visual examination of the distance between data points. In a similar manner, histograms are another helpful form of visualization for discerning outlier values. After identification, outlier values can be treated as missing values and dealt with using the aforementioned methods.

Data wrangling, sometimes also called data curation, refers to the process of improving existing data by correcting mistakes and merging data sets from different sources.^51^ Data cleaning covers the error correction part of the overarching data wrangling process and is arguably the most important part as well. When it comes to merging data sets, there are two major challenges. The first challenge is the lack of universal formatting. This can manifest as inconsistencies in the units or representations of similar variables. The second challenge is the potential lack of direct quantification. For example, suppose we apply machine learning on two sets of chemicals to tease out the relationship between their functional group composition and their solubility. One data set records the solubility in mass per volume (e.g., g/L), while the other uses molarity (e.g., mM). This is a case of a lack of universal formatting. To convert between the two units, the molecular weight is needed for each compound, which can be derived from structural information. When it comes to functional group composition, neither set of data provides the information directly. Instead, the chemical structures are represented with SMILES strings. This represents a lack of direct quantification. To obtain the functional group compositions, the SMILES strings need to be parsed with specialized programs or web tools to extract and quantify relevant structural information. Finally, the data can be merged once all relevant variables from both sets of data are quantified and represented in a uniform fashion. The merging process is simple. Concatenating one data set with another is usually sufficient. In the case where the ML algorithm may be sensitive to the order of the data, the concatenated set can then be scrambled to prevent the algorithm from learning the differences between the original data sets, instead of the differences between chemical compounds.

### Data Featurization

Now that different data types have been discussed, we can look at ways to acquire and process those data in order to make them usable for your ML model of interest. The process for acquiring usable data through converting non-numerical data into a numerical form is called data featurization. Chemical and biological data are not always presented as numbers, and since ML algorithms can only handle numbers as inputs, featurization is a necessary step before ML algorithms can be applied. These include chemical structures, DNA sequences, amino acid sequences, protein structures, protein–protein and protein–gene interactions, and so on. For chemical structures, the number of key functional groups can be counted and converted to a series of numbers with each number representing the number of a specific functional group. In addition, if certain numerical structural features, such as distance between atoms, bond angles, charges of atoms, etc., are of great importance to a prediction task, they can be directly included as input features as well.^52^ For DNA and amino acid sequences, since there are only a limited number of types of nucleotides and amino acids, they can be encoded as arrays of numbers to convert the sequence into numerical entries. For molecular interactions, logical true or false features or Boolean features denoting whether a pair of molecules is interacting can be concatenated to the list of input features. Distances between key atoms and functional groups belonging to different molecules can also be included as a feature when it comes to interactions.^52^ A popular framework for integrating chemical and biological information into ML is the so-called quantitative structure–activity relationship (QSAR). QSAR describes a general procedure for applying ML algorithms to experimental data and provides multiple methods to effectively select the relevant independent variables and convert them to numeric values.^53^

After featurization, the numerical representations of chemical and biological data can be further processed. A common procedure for refining data sets is called binning. It averages similar data points into single points, which reduces the overall noise in the data set. For example, when an image is converted into usable data, binning can be employed to reduce the amount of excess information and emphasize useful features of the image for the model. Specifically, image binning is accomplished by reducing the overall number of pixels by combining nearby pixels into a single pixel. In essence, this puts all of the data from pixels within a certain range into a singular bin and returns a single value. This has the benefit of reducing the overall noise in an image and ensuring that the model is trained only on the important parts when attempting to recognize a particular object or idea. Zhou described it well when he compared this process to attempting to train a model to recognize a leaf.^54^ If the only images shown to the machine are of leaves with serrated edges, the model could mistakenly believe that a true leaf is only one with serrated edges. This problem would be known as overfitting and will be discussed in more depth later on. Binning creates an elegant solution to this problem by decreasing the overall resolution of the image to a degree where it is still recognizable but any bias that has been accidentally introduced will be averaged out and go unnoticed by the model such as the serrated edges. Another way to think about this is in terms of controlling for extraneous variables in an experimental setup. Extraneous variables are any variables that you are not intending to research but can influence your dependent variable. As a result, these variables must be controlled to understand the true effect of the independent variable on the dependent variable; otherwise, false positive or negative results could afflict the outcome of your experiment.

An additional concept worth discussing for understanding how to process your data set is dimensionality. Dimensionality, or the number of dimensions, is a concept that most people are familiar with, even if it seems foreign in a mathematical sense. Dimensions are typically thought of in terms of physical space. For example, a cube has three dimensions as it contains length, width, and depth. In the same way, data can be visualized with a variety of dimensions. In its simplest form, the number of dimensions in your data set can be visualized by the number of axes on your graphical representation of your data or, in other words, the number of independent variables or features that you are analyzing. For example, a chemical data set represented with molecular weight, melting point, and number of aromatic rings has three dimensions. While a higher dimensionality will result in a more comprehensive representation of your data set, many ML models function best with a particular range of dimensions and may not function well or at all if the number of dimensions is outside that range. Additionally, even if the algorithm is capable of processing higher-dimensional data, the amount of computational time required may be so high that it renders the endeavor unviable.

**Figure 3:**
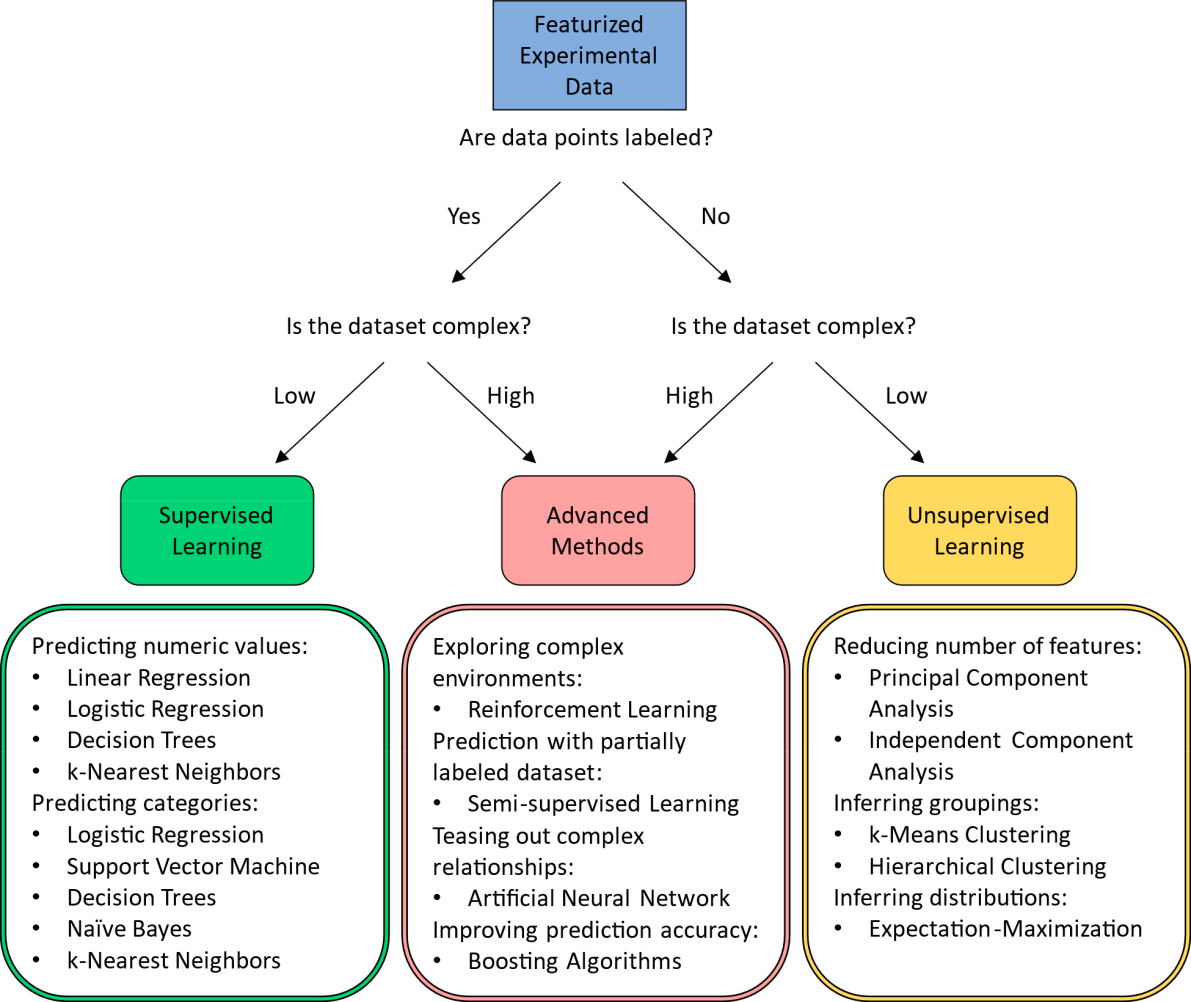
A general guideline to what type of machine learning algorithm to choose. Two things need to be considered: if the data set is labeled, and how complex is the data set. For complex data sets, advanced methods are usually preferable. For data sets with low complexity, if the data set is labeled, then supervised learning can be applied. If the data set is unlabeled, then unsupervised learning is applicable. Finally, each of the algorithms within the three categories has its own most suitable case, and the details can be found at the bottom of the figure.

When it comes to picking a suitable ML algorithm, the composition of your featurized data set is crucial. For a data set containing both features and labels, supervised learning is suitable for the task (Figure 3, left). For data sets with only features, unsupervised learning is capable of extracting information from them (Figure 3, right). For highly complex data sets, basic supervised or unsupervised learning algorithm would not be sufficient, and advanced methods are most suited for such tasks (Figure 3, middle).

### Data Normalization

Chemical and biological data span a wide range of magnitudes depending on the type of data. For example, the binding affinity of an enzyme to its substrate can be on the order of 1–100 nM, the bond angle of a specific pair of bonds can only take values between 0–360°, and the molecular weight of a molecule can go from 100 Da for small chemicals to over 10 000 Da for small proteins. Since most ML algorithms are only designed to handle raw numbers, the units and relative magnitudes between different types of data will be lost. In ML, the most common metric used to quantify the performance is in the form of the sum of the prediction errors. For example, if an ML algorithm is tasked to predict molecules with molecular weights of ∼500 Da as well as binding affinities of ∼50 nM, then the same 1% error rate would mean a ± 5 Da and a ± 0.5 nM difference, respectively. However, the algorithm sees only values 5 and 0.5. Since the goal of ML is to minimize its prediction error, the algorithm will be more likely to further reduce the error rate with respect to the molecular weight rather than the error rate with respect to the binding affinity. This may result in an undesired prioritization of one feature over another.

**Figure 4:**
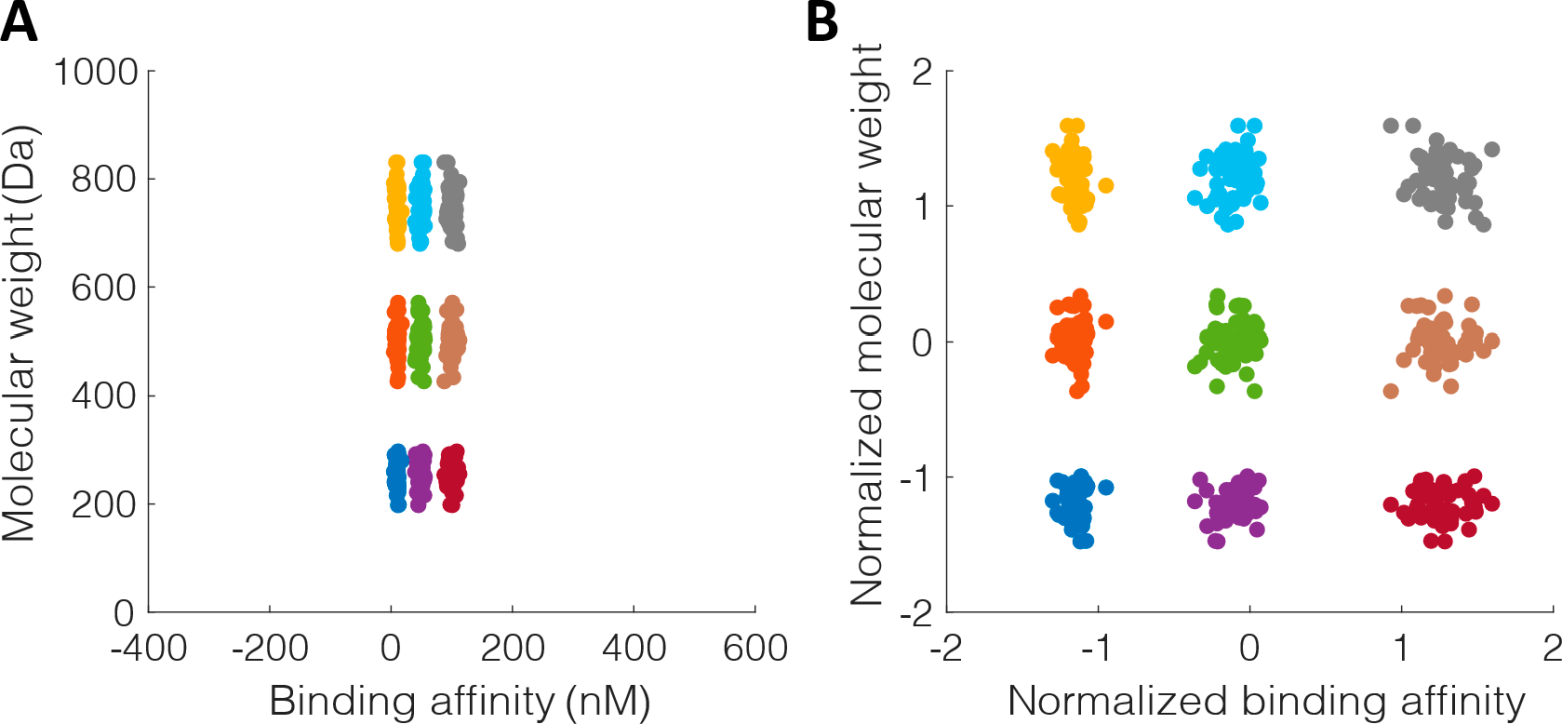
An example of data normalization using the standard score method. (A) For chemical and biological data, it is common when two features span across completely different numerical values. For example, a set of molecules may span across 150–850 Da in molecular weights, but only spanning 5–150 nM when their binding affinity to a target of interest is considered. ML algorithms do not inherently take units into consideration, which results in increased difficulty in discerning differences in binding affinities compared to molecular weights. (B) By using the standard score method, both binding affinity and molecular weight are normalized to a mean of 0 and a standard deviation of 1. Thus, the normalized values for both features now fall in between −2–2. By applying ML algorithms to the normalized values, both features will be equally prioritized.

To solve this problem, a technique called data normalization can be applied. The goal of data normalization is to rescale all variables into similar ranges so that the prediction errors will be on the same scale and thus be treated with equal importance by the ML algorithm. The most common normalization technique is called a standard score. It normalizes a given data set to have a mean of 0 and a standard deviation of 1. To carry out the normalization, the following equation can be applied to each feature:
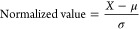


In the equation, *X* is the raw value of a data point, μ is the mean of the full data set, and σ is the standard deviation of the full data set. Another way to understand how normalization equalizes the importance of each feature is that it equalizes the distance between data points such that the similarities between data points are uniform across different features. As an example, suppose we have a data set of molecules weighing 150–850 Da, and their binding affinities to a target of interest range from 5–150 nM. Since ML algorithms treat them as raw numbers without units, the resulting scatter plot of this data set will look like Figure 4A, where the differences in binding affinities are much less prominent than those in molecular weights. After the standard score method is applied (Figure 4B), both molecular weights and binding affinities are standardized to between −2 and 2, enabling the ML algorithms to equally prioritize both features.

Data normalization has been proven to be one of the key steps in successfully applying ML algorithms to experimental data and can bridge the gap between data sets obtained using different technologies by rescaling them into the same range.^55,56^ Although some ML algorithms are capable of handling non-normalized data (an example being the decision tree algorithm), it is standard practice to normalize your data to avoid potential degradation in performance.

### Kernel Method

Many ML algorithms are designed to distinguish data points with large differences or, in other words, large distances between one another. They may perform poorly when two different categories of data points are too close to each other. However, due to the inherent randomness of biological and chemical processes, data produced by experiments may not present clear-cut boundaries between different types of data points. This would potentially hinder the learning processes of ML algorithms, even after proper data normalization. For example, chemicals derived from the same scaffold molecule may possess high structural similarities, but their biological activities can vary significantly from antagonistic to agonistic interactions. Similarly, many biological processes such as bacterial growth, receptor–ligand binding, and clearance rate of drugs are commonly used to gauge the effectiveness of therapeutics, but data recorded from these processes are often riddled with noise and uncertainties.

**Figure 5:**
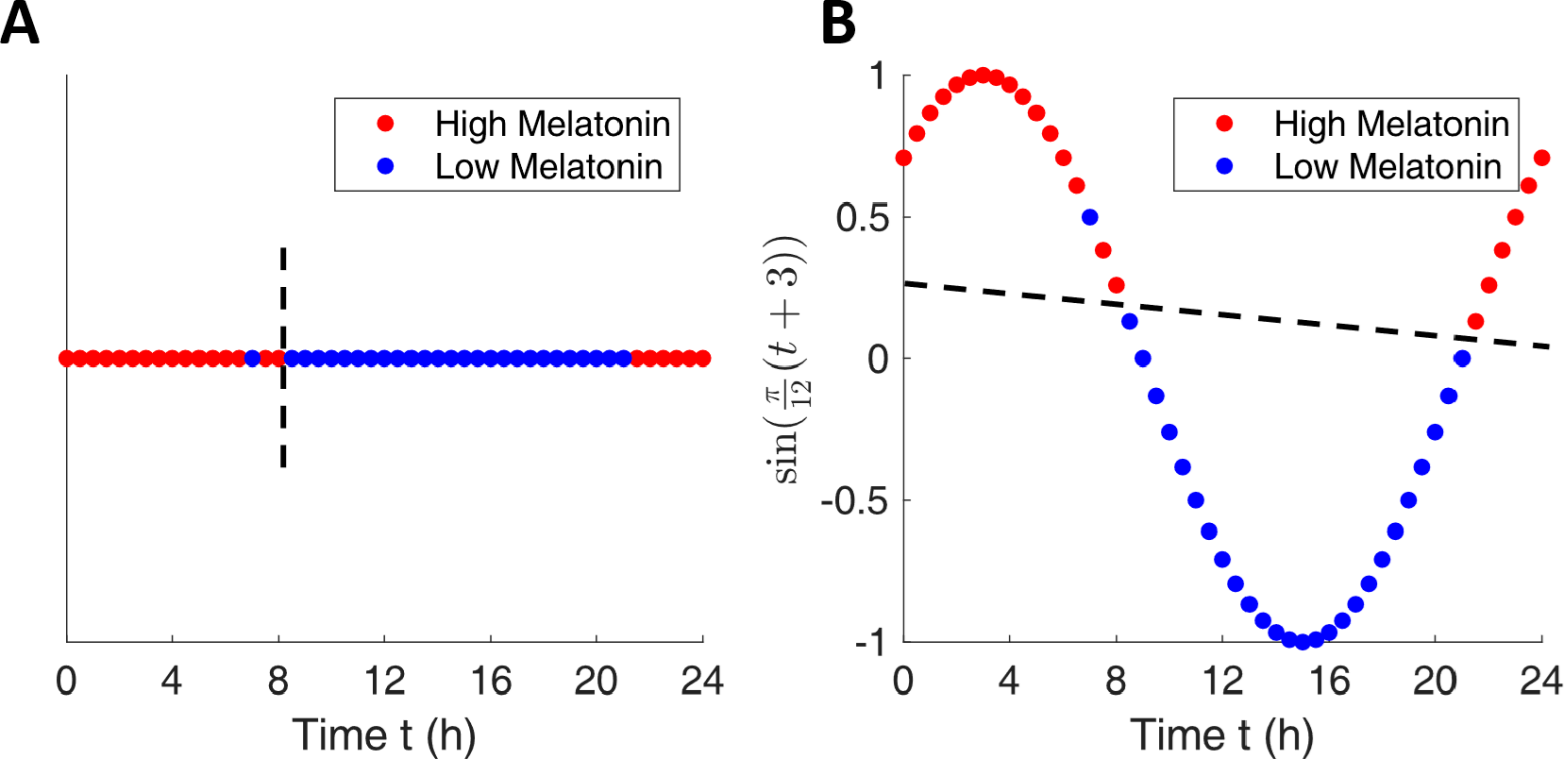
A simple application of the kernel method. (A) The raw data set consists of a binary label of melatonin level over a period of 24 h, corresponding to the amount of time spent in a day. The best singular straight cut of the data set to separate high melatonin points from low melatonin points is shown as a dashed vertical line, which results in a total of 7 mistakes (1 blue, 6 red). (B) A new 2D representation of the data set after the kernel method is applied. Combining the prior knowledge that blood melatonin level varies over the course of the day according to the circadian rhythm, and that melatonin level peaks around 3 AM, an artificial feature can be added by constructing a sinusoidal function with a period of 24 h and a peak at *t* = 3 h, and plugging the time values into the function. The new feature is plotted on the Y axis. With the new representation, the best singular straight cut to separate high melatonin levels from low levels is shown as the slanted dashed line. In this scenario, the best cut resulted in only 1 mistake (1 blue), which is a sharp decrease from the 7 mistakes in the previous panel.

One way to tackle this problem is to construct artificial features using functions or combinations of existing features to amplify the distance between crucial data points. Take melatonin levels as an example. Assume that we have a set of measurements of melatonin levels from an individual over the course of a day (Figure 5A). The measurements are classified as either high melatonin or low melatonin. Our goal here is to separate the data points into two groups with a singular, straight cut so that ideally one group will only contain points with high melatonin and the other only points with low melatonin. If we attempt to partition the data as is, then the best attempt we can make (dashed line, Figure 5A) will result in a total of 7 misclassified points, with 1 low melatonin point misclassified into the high melatonin group (near *t* = 7 h), and 6 high melatonin points misclassified into the low melatonin group (for *t* > 21 h). However, the performance could be improved. According to past research, blood melatonin levels are dependent on the circadian rhythm, with a period close to 24 h. These levels also peak at around 3:00 AM (or t = 3 h) and dip during the day, resembling a sinusoidal function. Thus, we can construct an artificial feature with a sinusoidal function with a period of 24 h and a peak at *t* = 3 h to incorporate the periodic nature of blood melatonin levels. Mathematically, this sinusoidal function is represented as . By plugging the time into the sinusoidal function, the artificial feature (plotted on the Y axis in Figure 5B) amplifies the distance between data points around *t* = 8 h and *t* = 20 h that are crucial for accurately separating the high melatonin points and the low melatonin points. Now, if we attempt to classify the data again, we can easily separate the data in a linear fashion (dashed line, Figure 5B), with only one low melatonin point misclassified as the high melatonin group. The act of introducing artificial features that are functions or combinations of existing features is called the kernel method.^57^

Since the goal of introducing artificial features is to increase the distance between data points, it has been proven mathematically that this process is equivalent to using a custom-made function to calculate the distance. The distance function used in ML algorithms is commonly called the kernel function. The most intuitive function is the Euclidean distance function, which is defined as
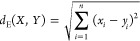


In this equation, *d*_E_(*X*,*Y*) is the Euclidean distance between points *X* and *Y*, *x*_*i*_ and *y*_*i*_ represent the *i*th feature of *X* and *Y*, and *n* is the number of different types of features in the data set. The Euclidean distance is intuitive in that it coincides with our sense of distance in the physical world, but when it comes to ML, the Euclidean distance does not always provide the largest distinction between data points. Another widely applied kernel function is called the radial basis function (RBF) kernel. It is defined as follows:
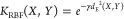


In this equation, *K*_RBF_(*X*,*Y*) is the value calculated by the RBF kernel, *e* is Euler’s number, γ is a free positive parameter, and *d*_E_(*X*,*Y*) is the Euclidean distance between points *X* and *Y*. The parameter γ determines how fast *K*_RBF_ decays as the *d*_E_(*X*,*Y*) increases. Due to the presence of an exponential function and a nonpositive exponent, the RBF kernel always returns a number between 0 and 1, reaching 1 when *X* is identical to *Y*, and decreasing in value as the Euclidean distance between *X* and *Y* increases. In this sense, *K*_RBF_ is a measurement of the similarity between two given points. The limited range of the function output also simplifies the calculation in downstream ML algorithms.

To determine the type of kernel to use, prior knowledge on the field of study or observations on the data set needs to be obtained. Additional kernel functions to the Euclidian distance function and the radial basis function include the polynomial kernel, sigmoid kernel, and Gaussian kernel. The details of these functions are out of the scope of this review, but another review written by Vert and Jacob covers kernel methods in more detail.^58^

### Small or Biased Data Sets

Chemical and biological data are inherently difficult to acquire due to the high complexity and low scalability of the experiments. In addition, when screening for biological activities, the hit rate for library-based screens is typically low, sometimes even less than 1%. Thus, it is a common occurrence when applying ML to these data sets that there are simply not enough positive data points to efficiently train an unbiased model. As ML has developed through the past decades, algorithms specializing in data-scarce regimes have come to exist, which include one-shot learning and transfer learning. However, there is a much simpler solution to this problem: a statistical technique called bootstrapping. Bootstrapping treats the input data set as a new population and repeatedly samples it randomly with replacement to generate a new data set. For example, for an input data set of 3 elements denoted as the set {*x*_1_,*x*_2_,*x*_3_}, a bootstrap with 2 elements per sample and 5 total samples can be {*x*_1_,*x*_2_}, {*x*_2_,*x*_3_}, {*x*_2_,*x*_2_}, {*x*_2_,*x*_3_}, {*x*_3_,*x*_3_}. This results in a new data set with more data points than the original data set, while ensuring the new data set still conforms to the distribution of the true population where the original data set comes from. Bootstrapping can be applied to any ML algorithm when the input data set is too small or too biased to obtain a meaningful model.

An example of bootstrapping can be found in the following research on the side effects of various drugs on heart function, conducted by Sun et al.^59^ The authors generated a support vector machine (SVM, discussed in greater detail in later sections) model to predict whether a given drug will inhibit the potassium ion channel protein encoded by the human ether-à-go-go-related gene (hERG). Inhibition of hERG can cause arrhythmia that can be life threatening, and such inhibition has been the cause of the withdrawal of many extremely popular and promising drugs in the past. The authors utilized a high throughput screening modality to generate a labeled data set of 3,024 compounds. However, only ∼16% were identified as hERG blockers, resulting in a significant bias against hERG blocking activities. To compensate for such bias, the authors constructed 5 sets of bootstrapped data, with each containing 10, 20, 30, 40, or 50 random subsets of the majority class (drugs that do not block hERG). For each subset, the number of samples is equal to the number of data points of the minority class (hERG blockers). The resulting models achieved high performance, quantified by a high true positive rate together with a low false positive rate. The authors also note that significant improvement in performance was seen when comparing the 20-subset bootstrapped data set to the 10-subset one, but no significant improvements were seen comparing the bootstrapped data set with 20–40 subsets.

Now that we have discussed the properties of your potential data sets, we can move on to discuss the ML models that will assist in analyzing and forming predictions from that data.

## Supervised Learning

The task of supervised ML algorithms is to construct a mathematical model to infer a relationship between the features (or inputs, including but not limited to the chemical structure of a compound, its molecular weight, functional group composition, and similarity to known compounds) and the labels (or outputs, such as activity against a protein of interest, inhibition or promotion of cell growth, side effects on bystander cells, etc.).^60^ This process is commonly referred to as training. The model’s performance is then tested using a separate data set, containing both features and labels, that was not utilized in the training process. Due to the difficulty of obtaining multiple data sets, a common practice is to split one data set into a training set and a testing set. Finally, the model is applied to data with only features to predict their labels. This process is called supervised learning because it requires prelabeled data, most likely labeled by humans, to conduct the training process. Supervised learning algorithms are capable of both regression (predicting a continuous variable) and classification (predicting a discrete variable).^60^ In this section, seven supervised learning algorithms widely used in small molecule development are introduced, each with its own advantages and disadvantages.

### Linear Regression

Linear regression is one of the most basic supervised learning algorithms. In its simplest form, linear regression seeks to find the relationship between the independent variable and the dependent variable. It accomplishes this by plotting the points associated with the variables before finding a straight line that approximates the points. The line can then be quantified by a linear equation. Linear regression is most commonly used for quantitative variables, either continuous or discrete. If either the independent variable or the dependent variable is qualitative, linear regression can still be performed by assigning numerical values to the categories, but its performance tends to be lower than that of other algorithms designed to handle qualitative data. Because of its simplicity and approachability, linear regression has been applied across a wide variety of fields and serves as the first-line method for interpreting data sets.

In ML contexts, the linear regression model uses a linear combination of all of the input features to approximate the labels. It most commonly utilizes the method of least-squares to find the best linear equation that fits the data. In other words, it attempts to minimize the sum of the squared distances from the training data to the linear equation, which represents the error of the chosen line. This function is termed the loss function, as it represents the amount of information that will be lost if the training data are replaced by the linear equation. This form of linear regression was first used by the French mathematician Adrien-Marie Legendre and German mathematician Carl Friedrich Gauss in the early 1800s to predict planetary movements^61^ and has seen extensive developments in the two centuries that followed. A closed-form solution, i.e. a deterministic formula, for the parameters of linear regressions with any number of features, was developed some 200 years ago, but the practicality of the solution to real-world data is questionable because its computational complexity increases exponentially as more features are included.^62^ An alternative method, called gradient descent, gradually changes the parameter in a stepwise manner, such that the loss function continues to decrease for each step. Gradient descent requires much less computational power than computing the closed-form solution when it comes to large data sets. However, it may result in solutions that are optimal when compared to similar ones (i.e., a “local” optimum) but suboptimal when compared against all possible solutions (i.e., failing to find the “global” optimum). This can be circumvented by running multiple rounds of gradient descent with randomized initial parameters and selecting the result with the minimum loss.^62^

With the computational power available today, linear regressions are easy to perform and can be used as a quick and simple method to verify dependencies between labels and features. The fact that only linear dependencies can be assumed means the results of linear regressions are easy to interpret.^63^ However, it is also limited by the same assumption, in that it cannot readily generalize to complex data sets that cannot be approximated as a linear function. To remedy this shortcoming, the kernel method can be applied to provide an extension to nonlinear relationships.

An example of linear regression can be seen in the 2021 study by Janairo et al., in which they utilized a multiple linear regression (MLR) model to predict the binding free energy of potential protease inhibitors of SARS-CoV-2.^64^ MLR is a simple combination of multiple regular linear regression models. It predicts multiple labels using the same set of features. The researchers compared MLR to a variety of other model types to answer this question but found that MLR outperformed these other models, despite being the simplest model. The MLR model in this study was able to avoid overfitting and was the most consistent of the models tested, as it showed a significantly better fit of the data to the model (quantified by the correlation coefficient, or *r*^2^ value) and a much lower prediction error (quantified by root–mean–square error, or RMSE). The authors also valued the interpretability of the MLR model compared to the other methods, as MLR allows for a greater understanding and explainability of the internal methodology used by the model when compared to other model types that may utilize a more “black box” approach. This resultant model was able to predict the binding affinity of the potential protease inhibitors with greater than 70% accuracy and was validated using molecular docking. This study shows the utility of MLR models and highlights the importance of avoiding the assumption that a more complex model is guaranteed to perform better on a given data set. Additionally, experiments such as this could help reduce the number of compounds for experimental testing and propel the discovery of therapeutic molecules forward by eliminating a large number of compounds by testing them in silico prior to experimental testing.

### Logistic Regression

Logistic regression is a model that, like linear regression, seeks to find relationships between variables and make predictions from the observed relationships. However, instead of requiring those relationships to be linear, a logistic (sigmoid) model is used. Since the sigmoid function produces any number bounded between 0 and 1, the resulting prediction can be interpreted either as a continuous label or the probability of a discrete label. This allows the model to handle data that is not continuous, and as a result, logistic regression is quite adept at handling qualitative data. The independent variable can be either qualitative or quantitative, but the dependent variable must be qualitative for the model to function properly. As a result, it is possible for the dependent variable to be made of binary, nominal, or ordinal data. However, one should avoid attempting to fit a data set that is too small or made up of more features than data points, as this will result in a very poor predictive ability.

Many aspects of the training process of logistic regression remain the same as linear regression, including the use of the sum of squared distances as the loss function and gradient descent as a tool to minimize the loss function. Unlike linear functions, sigmoid functions are nonlinear by nature. Since sigmoid functions appear frequently in biological contexts, such as dose response, cell proliferation with limited nutrients, and ligand/receptor binding kinetics, logistic regression has proven to be useful in the field of small molecule design. In these scenarios, logistic regression can be readily applied and the results easily interpreted. In addition to these applications, the fact that the sigmoid curve spans only between 0 and 1 allows for its alternative interpretation as a probability. The most common application of this interpretation is to model the log-odds of observing a label, defined as , where *p* represents the probability of observing said label. Thus, logistic regression can also be applied to classification tasks where discrete, categorical, or qualitative labels are predicted in addition to traditional regression tasks that output continuous labels. Finally, like linear regression, logistic regression is computationally light and easy to calculate and is well suited for initial investigations of data sets. However, since not all nonlinear relationships follow the sigmoidal function, it is always preferable to first manually check if the data follow a sigmoidal function and then apply logistic regression.

An example of logistic regression can be seen in the 2014 paper by Gfeller et al., in which the researchers apply a logistic regression model to assess the bioactivity of small molecules from their structures.^65^ The categorical nature of structural data makes logistic regression an ideal choice for this kind of problem. The authors based their model on the similarity of a query molecule to those within the training data set, which is assessed through the comparison of their molecular fingerprints and 3D spatial structures. The model is publicly accessible and can be utilized by researchers to prescreen potential bioactive molecules, thus saving valuable time and costs on experimental screening. This tool is called SwissTargetPrediction, and it is uniquely capable of taking into account both the 2D and 3D structures of the target molecules. These structures are assessed using logistic regression to return a prediction of the bioactivity for that molecule. The model was recently updated in 2019 by Daina et al., and it was capable of predicting at least 1 correct target molecule among the top 15 predictions for over 70% of the compounds tested with this model.^66^

### Support Vector Machine (SVM)

A support vector machine (SVM) is a model that discerns the relationship of a number of independent variables to a dependent variable. These variables can be made up of qualitative or quantitative data that are either discrete or continuous. As a result, it is capable of being utilized for either classification or regression problems, which makes it a versatile tool.^67^ However, the utility of SVM is limited by its inability to handle exceptionally large data sets or those with a great deal of overlap between features. Since SVM is more commonly used for classification problems, the inner workings of SVM for classification will be expanded upon in this section.

For classification tasks, SVM utilizes an algorithm to generate a hyperplane to separate the data into two categories. This process is akin to slicing a pizza with two toppings on different sides into two parts while minimizing the crossover of the toppings. A hyperplane is a linear object that serves to extend 2D lines and 3D planes to higher dimensions. This hyperplane divides the space occupied by the input features into two sides and is generated by minimizing the amount of crossover of the two labels according to the training data. After training, the same hyperplane is used to predict the labels of unlabeled data points based on their location on either side of the hyperplane. Due to its ability to segment the feature space, SVM is highly suitable for biological labels that are more likely to be discrete categories than continuous numbers, and has been frequently used for genetic and other biological data.^67^ In addition, the kernel method is still applicable to SVM, enabling the algorithm to effectively adapt to complex feature spaces. This allows for higher specificity when separating samples that may otherwise have been found as outliers on the wrong side of the hyperplane.^60^ One drawback of this method emerges from the hyperplane itself, which is inherently limited to binary segmentation. However, this limitation can be overcome through training multiple SVMs for data sets with more than two categories. First, one category is chosen, and an SVM is trained to predict whether the training data points belong to this category or not. Then, a second category other than the first one is chosen and an SVM is generated to predict if the training data points belong to the second category or not. Repeat this process for all remaining categories to produce a full segmentation of the whole data set.

An example of an SVM can be seen in the 2017 paper by Chen and Visco, in which they utilized SVM models to screen the PubChem Compound Database to identify compounds with the potential to inhibit the Cathepsin L receptor.^68^ The Cathepsin L receptor is thought to be a key receptor in many viral disease pathways, including malaria and Ebola. The researchers used SVM both to classify their data into an initial active/inactive data set and then again to perform a regression to identify the strength of the activity for each compound. SVM was an ideal fit for this problem due to its ability to perform both classification and regression, which helped to narrow down their data set and expedite inhibitor identification. This study shows the potential for significantly reducing the cost of ligand discovery by implementing a first round of testing in silico. Following this, the predictions were experimentally validated, with the results being used to further refine their model to obtain a final predictive accuracy of 75%. This approach significantly improved upon the efficiency of previous screening methods, with traditional high-throughput screening methods typically only reaching a success rate of <1%.

### Decision Trees

A decision tree is an extremely straightforward method of ML in which a number of independent variables, in the form of features, are used to create a branching path that leads you toward your dependent variable with increasing specificity as you proceed along the tree. The variables can be either quantitative or qualitative, and the quantitative variables can be continuous or discrete. Each split along the branching path is performed on a single independent variable, which results in this model being ideal for experiments with a large number of independent variables. However, a single tree can predict only one dependent variable, so multiple trees need to be generated for more than one dependent variable. Additionally, decision trees do not perform well on large data sets, as it would be overly computationally demanding.

The decision tree is a common method of supervised ML, which employs a hierarchical decision-making model to split input features according to the labels in the training data.^60^ This method is fast to calculate and can utilize both numerical and categorical data. The tree itself is compiled through a successive binary splitting of the input features while minimizing the prediction error for each split by comparing the performance of all possible splits. Thus, the resulting decision tree can also be visualized as a flowchart (such as Figure 3) for ease of rational interpretation. The output of a decision tree is in the form of discrete categories. Since the predictions are not derived from a linear combination of the input features, decision trees are inherently capable of handling nonlinear data sets. However, decisions made by a decision tree are not necessarily infallible and are prone to overfitting due to the nature of categorization: with a large tree depth, or a larger number of consecutive decisions, categories run the risk of containing very few data points, leading to overfitting and poor prediction performance on testing data.^60^ In addition, decision trees are not the best choice for predictions of numerical labels due to the categorical nature of its output, which can result in lower accuracy when compared with other models trained with the same data. However, continuous labels can be predicted by taking the average of the labels in the predicted category. Despite these drawbacks, the performance of this model can be improved by generating multiple decision trees on the same data set. This method is called the random forest algorithm and will be covered in detail in the next section.

**Figure 6:**
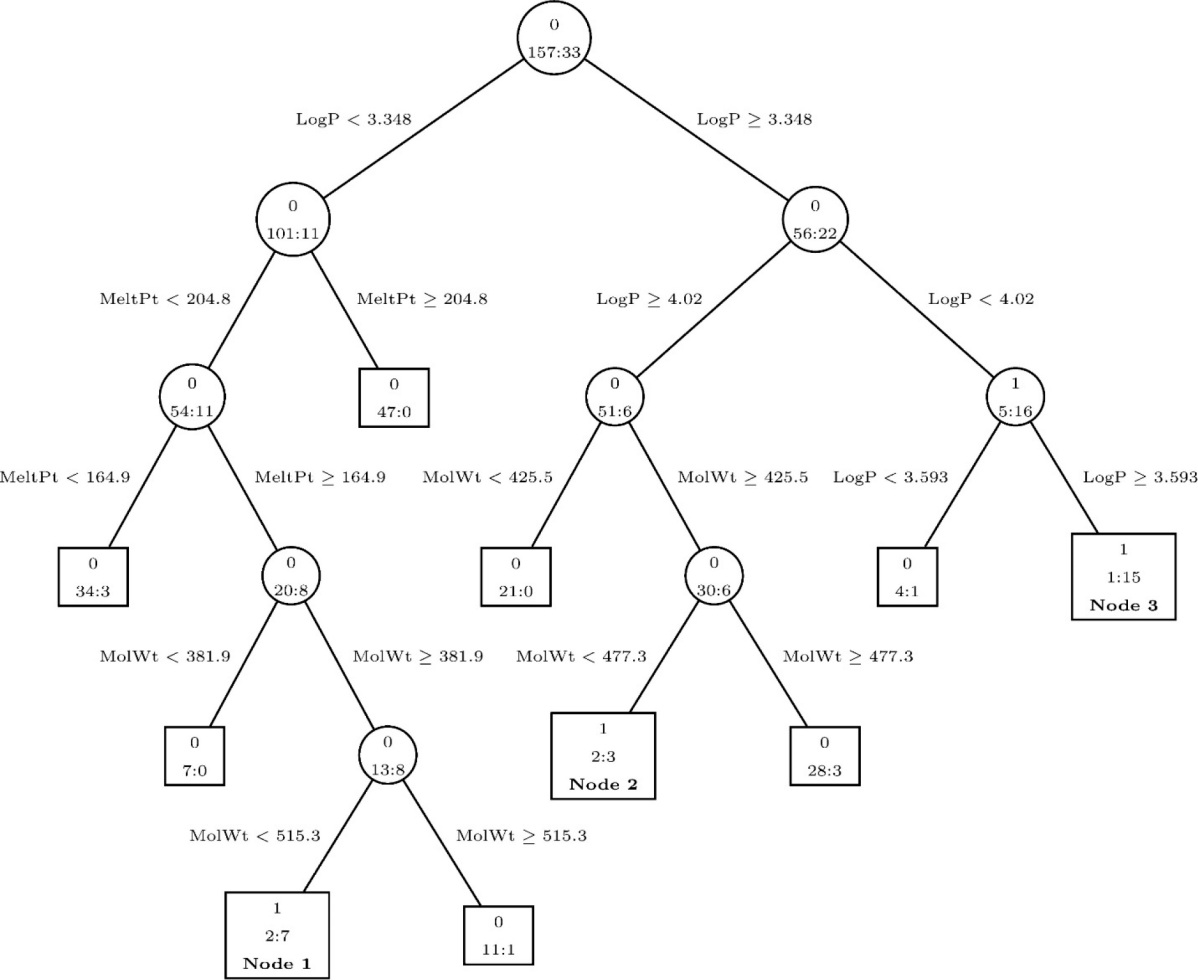
Decision tree model created for high-throughput drug discovery. The circular nodes are those that lead to a subsequent node, while the square nodes are those that end in a terminal decision. This model utilizes common molecular dimensions such as molecular weight (MolWt) and melting point (MeltPt). The decision for this model is binary, resulting in either inactive (0) or active (1), which is displayed in the top row of each node. The total number of samples is displayed in the second row of each node to the left of the colons, with the number of active compounds yet to be detected on the other side of the colons. Those that are labeled as nodes 1, 2, or 3 are the only nodes that result in the classification of the molecules as active. Reproduced from ref (69). Copyright 2012 American Chemical Society.

An example of a typical decision tree model can be seen in Figure 6, which is derived from a study by Yuan et al.^69^ The authors of this paper utilized both a decision tree and a “tree harvesting” algorithm to improve the accuracy of the tree. They only analyzed simulated data, but through that they were able to demonstrate the applicability of the decision tree method to the processing of chemical discovery data. As can be seen in Figure 6, the tree that they generated contained nodes for making decisions (first row of number on the nodes, 0 as inactive, and 1 as active) based on common chemical properties such as the melting point (MeltPt) and molecular weight (MolWt). The total number of chemicals in a node is shown to the left of the colon on the second row of each node, while the number of positive chemicals yet to be classified appears on the other side of the colon. A similar approach can be applied to real data to help narrow down the molecular possibilities and make subsequent screening attempts more efficient.

### Random Forest

The random forest algorithm, initially formulated in 1995, constructs an ensemble of multiple decision tree models.^70^ The motivation for this method arose from the problem that decision trees tend to overfit the training data due to its prediction being an average of a subset of the training data. By constructing multiple decision trees, this ensemble model will potentially achieve higher performance, while the computational cost remains relatively low due to the ease of generating decision trees.

However, there is another layer of complication when it comes to constructing multiple decision trees on the same data set: the decision tree algorithm is deterministic, and without alterations to the input, the model will remain the same. To overcome this problem, bootstrapping can be employed. Utilizing bootstrapping, it is possible to generate different bootstrapped data sets from the same input data set and train multiple unique decision trees. The final prediction can be calculated as the average of predictions from all decision trees for continuous labels or determined to be the most commonly predicted label for categorical labels, also known as plurality voting. This has proven to vastly reduce overfitting compared with single decision tree models. To further reduce overfitting, a random selection of input features can be artificially removed or hidden during training of each decision tree. This is to prevent the case where all models converge onto using one or a few input features when these features are much more correlated with the labels than the rest of the features.

An example of a random forest classification can be seen in the 2021 experiment from Kapsiani and Howlin in which they were able to predict whether a compound would extend the life of *C. elegans*.^71^ Using molecular descriptors as their features from the DrugAge database, the authors trained 5 random forest models on compounds with confirmed antiaging abilities. The best model achieved a prediction accuracy of 85.3%, and was further applied to an external database consisting of 1,738 small molecule compounds, where 15 compounds were predicted to extend the life of *C. elegans* with over 80% predicted probability. Though the study lacked experimental validation on its own, 9 out of the 15 predicted compounds were validated from the previous literature.

### Naïve Bayes Algorithm

The naïve Bayes algorithm is similar to SVM and decision trees in that it is designed for classification tasks. As a result, the best outcome will be from the use of qualitative variables for the variables, unless the quantitative variables are normally distributed.^60^ However, in contrast to SVM and decision trees only being able to return a single label as the output, naïve Bayes algorithm produces a comprehensive list of probabilities for observing each possible label value.^72^ It achieves this by employing Bayes’ theorem to predict the probability of each label according to the input features. For example, suppose we would like to predict the incidence rate, or probability of cancer in the population over the age of 80, denoted as P(Cancer | Age >80), also called the posterior. Bayes’ theorem states that the posterior probability can be calculated as
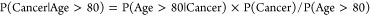


P(Age >80 | Cancer), also called the likelihood, denotes the probability of observing people over the age of 80 within the population that has been diagnosed with cancer. P(Cancer), or the prior, is the probability of observing cancer patients within the entire population. Similarly, P(Age >80), or the evidence, is the probability of observing people of age over 80 in the entire population. Assuming we sampled 500 random individuals in the population, a possible result can be seen in Table 1.

**Table 1:** A Simple Example Data Set to Illustrate Bayes’ Theorem

	Cancer	No Cancer	Total
Age > 80	4	46	50 (10%)
Age ≤ 80	1	449	450 (90%)
Total	5 (1%)	495 (99%)	500

In this case, the likelihood is
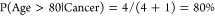


Thus, the posterior can be calculated as follows:
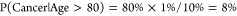


The naïve Bayes algorithm applies the same rule to a labeled input data set. The features take the place of the “age” criteria in the example, and the labels take the place of the “cancer” criteria in the sample. For categorical data, the algorithm calculates the posterior probability P(label | features) for each label in the same way as the aforementioned tabulated example. For continuous data, a probability distribution needs to be assumed for each of the features in order to allow the query of any value on each of the features. A common distribution is the Gaussian distribution, also known as a normal distribution. Each distribution contains parameters that need to be fine-tuned to fit the input features. Then the algorithm again applies Bayes’ theorem to solve for the posterior probability. Here, a key assumption is made: all input features are mutually independent of each other. That is, changes in one feature do not result in changes in any of the other features. With this naïve assumption, the posterior probability is proportional to the product of the likelihood for each feature P(feature | label), and the prior probability for each label P(label). Since the evidence P(feature) is only dependent on the composition of the input data set and not the fitted distribution, it becomes a constant scaling factor and does not need to be optimized. Finally, to derive the parameters of the distributions, the method of maximum likelihood is applied, where the parameters are gradually tuned to maximize the posterior probability.

An example of a naïve Bayes model can be seen in the 2018 paper by Perryman et al. in which the researchers constructed a model for predicting the potential cytotoxicity of experimental therapeutic compounds on Vero cells.^73^ They obtained their training data from molecules that had been previously assayed for their cytotoxicity. The training data set contains a variety of features, including molecular weight, number of aromatic rings, number of hydrogen bond donors/acceptors, and so on. The naïve Bayes model trained on the aforementioned data set was able to predict cytotoxic effects and was validated on molecules outside of the training data set with known properties. In a later publication from the same research group, a similar methodology was utilized to prescreen compounds that could be used against *Rickettsia canadensis* infections on a Bayesian model prior to experimental high throughput screening.^74^ This latter experiment validated the utility of naïve Bayes models in experimental settings. The training set for the former model was made available to experimentalists interested in prescreening the cytotoxic effects of molecules and could prove highly beneficial in reducing the number of molecules needing experimental screening, thus accelerating the rate of therapeutic molecule development.

### *k*-Nearest Neighbors (*k*-NN)

The *k*-nearest neighbors (*k*-NN) algorithm is a flexible algorithm that can predict both discrete and continuous labels. The variables can be either quantitative or qualitative, and the quantitative variables can be either continuous or discrete. The variables from the training data set are mapped as points on a graph, with the labels of training points being used to predict the labels of unlabeled points outside of the training data.^75^ This makes *k*-NN extremely useful for data sets with missing labels, where labeled points can be used as the training data set for *k*-NN to generate labels for points without one. However, limitations do exist, as *k*-NN cannot work effectively on large data sets. This is because the number of dimensions increases as the number of features in the data set increases, which renders the algorithm ineffective. Each additional dimension added here serves to further separate the data until it becomes difficult for the algorithm to discern the relatedness between the data points due to their vast separation.

**Figure 7:**
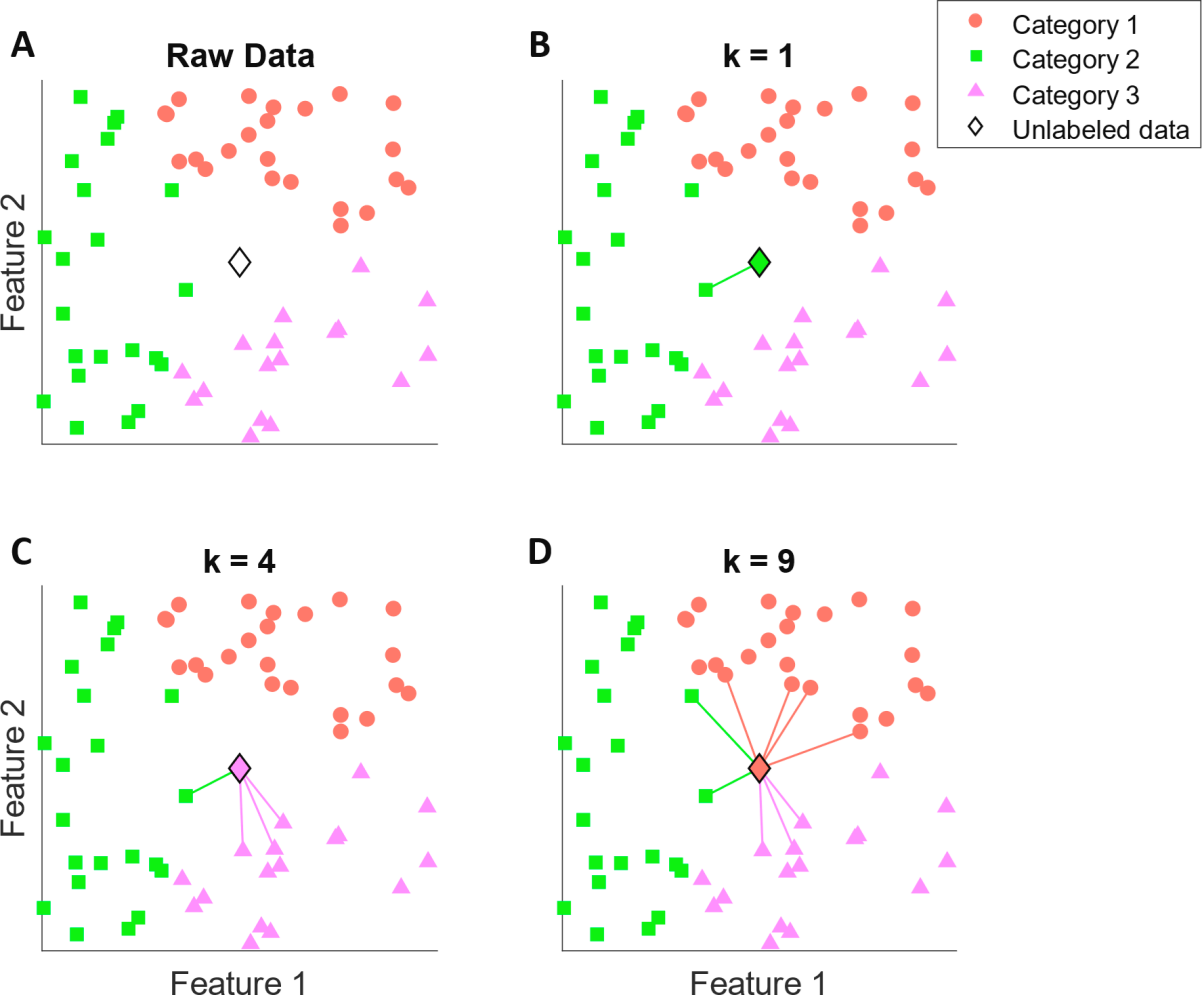
Visualization of the *k*-nearest neighbor (*k*-NN) algorithm. (A) A labeled data set with 2 features and 3 categories (orange circles, green squares, pink triangles). An additional unlabeled data point is located at the center (black diamond outline). (B) The *k*-NN algorithm is applied to determine the category of the unlabeled data point. With *k* = 1, only the nearest labeled point to the unlabeled point is considered, indicated by a line between the two points. In this case, the nearest labeled point belongs to category 2, and thus the unlabeled point is assigned to category 2 as well (green diamond). (C) With *k* = 4, there are a total of 1 point in category 2 and 3 points in category 3 near the unlabeled point. By applying plurality voting (3 > 1), the unlabeled point is assigned to category 3 (pink diamond). (D) With *k* = 9, there are a total of 4 points in category 1, 2 points in category 2, and 3 points in category 3 near the unlabeled point. Through plurality voting (4 > 3 > 2), the unlabeled point is assigned to category 1 (orange diamond).

The core concept of *k*-NN is simple: points close to one another should have similar labels. The algorithm is quite distinct from the previous supervised learning algorithms in that it does not require training. When given a labeled data set, and an unlabeled point with a set of features to predict its label, *k*-NN first picks *k* labeled data points closest to, or the most similar to, the unlabeled point in terms of their features. To derive a discrete label, the algorithm applies plurality voting from the selected labeled points, which means that the point of interest is assigned the label that is most common among the *k* closest labeled points (Figure 7). To derive a continuous label, the algorithm averages the labels of the selected labeled points. A major benefit of *k*-NN is its ability to classify more than two categories without needing multiple models, which is due to the nature of plurality voting. In addition, the fact that *k*-NN does not require training means that it is easy to implement. However, *k*-NN will produce highly different results depending on the type of kernel function chosen. To remedy this drawback, multiple *k*-NN models with different kernel functions that emphasize different features can be tested and selected for best performance. Different *k* values should also be tested, since with larger *k* values, the prediction will be based on a more comprehensive selection of points, but you run the risk of including points too dissimilar to the point of interest and vice versa. This issue is demonstrated in Figure 7 where the point of interest is situated at distances similar to three categories of labeled data points (Figure 7A). With *k* = 1, the point of interest will be labeled according to the single nearest point, which belongs to category 2 (Figure 7B). With *k* = 4, the 4 nearest points are considered for labeling the point of interest. Since there are 3 points in category 3 and 1 point in category 2, the point of interest is labeled as category 3 (Figure 7C). In a similar manner, with *k* = 9, the point of interest is labeled as category 1 since within the 9 nearest points there are 4 points belonging to category 1, a number higher than both categories 2 and 3 (Figure 7D). Another drawback lies in the amount of calculation needed. As the labeled data set increases in size, the amount of calculation increases rapidly since each prediction requires as many similarity calculations as the number of the labeled data points.

An example of *k*-NN can be seen in the 2020 paper by Arian et al., in which the authors utilized a *k*-NN model to identify small molecules capable of inhibiting protein kinases that play important roles in cancer.^76^ The researchers selected a set of six molecular descriptors as features before using *k*-NN to classify unknown molecules into those capable of inhibiting kinases and those incapable. They utilized seven neighbors for determining the unknown molecules. The model was validated through comparison with SVM and naïve Bayes algorithm, with the *k*-NN model outperforming the others on all metrics, including accuracy (percentage of correct predictions among all predictions), sensitivity (percentage of correct predictions among all molecules experimentally verified to inhibit protein kinases), and specificity (percentage of correct predictions among all molecules experimentally verified to not inhibit protein kinases).

### Summary

**Figure 8:**
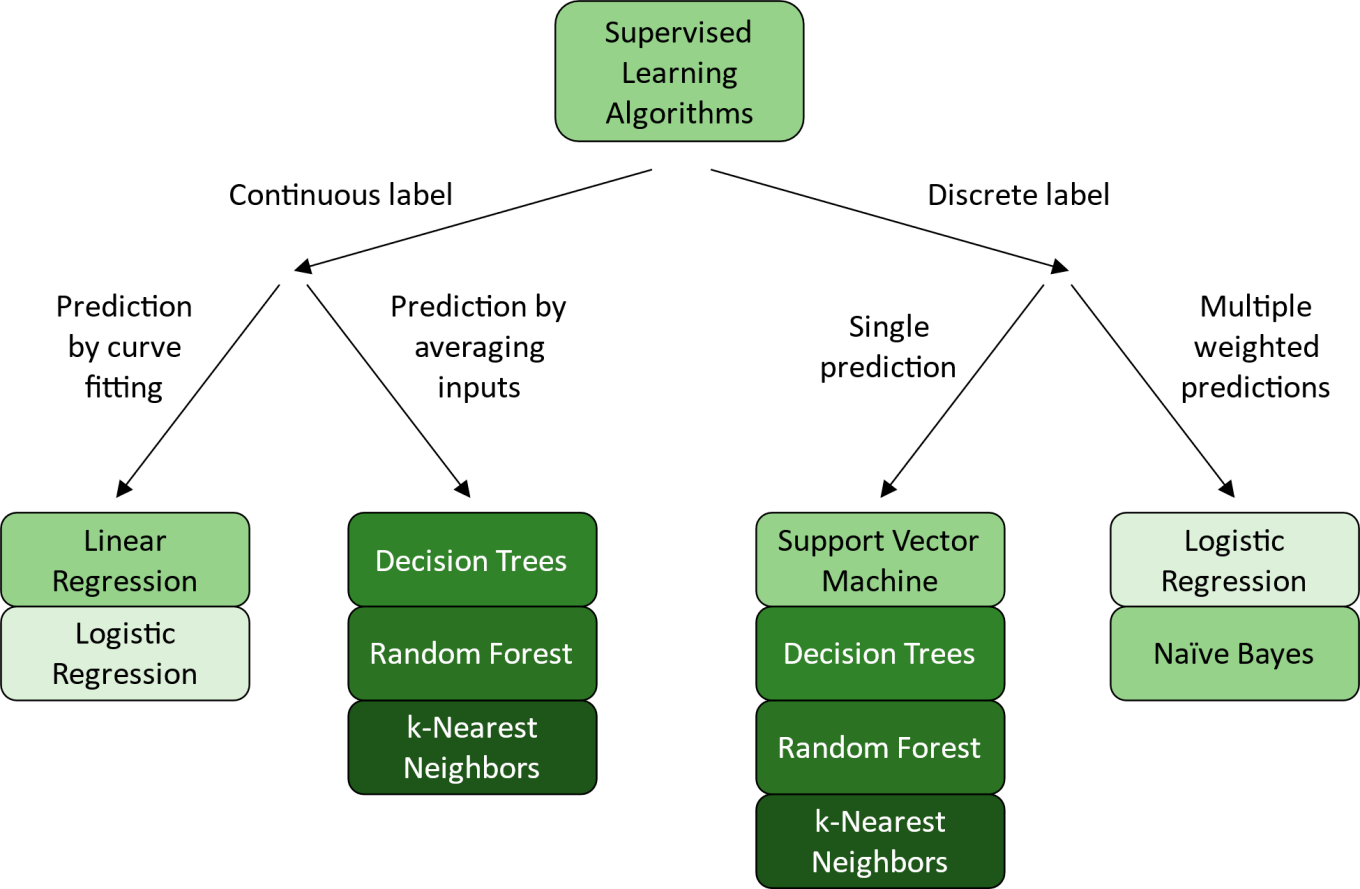
A simple diagram for determining what supervised learning algorithms to use. In this review, a total of 6 supervised learning algorithms are introduced, each with its own strength and weaknesses. To determine which algorithm suits your needs, first the type of label needs to be considered. For continuous labels, there are 5 algorithms suitable for such predictions. Linear regression and logistic regression predict continuous labels by performing a curve fitting on the full input data set, while decision trees, random forest, and *k*-nearest neighbors achieve this through averaging a subset of input data points. For discrete labels, including qualitative and categorical labels, there are 6 algorithms to choose from. Support vector machine, decision trees, random forest, and *k*-nearest neighbors will make a single prediction on the most suitable label, while logistic regression and naïve Bayes generate a comprehensive list of possible labels, each with a probability or weight attached to it. Of note, logistic regression, decision trees, and *k*-nearest neighbors are capable of predicting both continuous and discrete labels.

In this section, seven distinct supervised learning algorithms were introduced and explained. They are all designed to make predictions using training data sets composed of both features and labels, but depending on the type of predicted label, the nature of the algorithm, and the type of the output, different algorithms will suit different needs (Figure 8). Some methods can handle both continuous and discrete labels, such as logistic regression, decision trees, random forest, and *k*-nearest neighbors, while others can only handle one or the other, such as linear regression, support vector machine, and naïve Bayes. For continuous label prediction (Figure 8, left), linear regression and logistic regression predict the labels by fitting a curve to the training data, while decision trees, random forest, and *k*-nearest neighbors predict the labels by averaging a small group of training data points. For discrete label prediction (Figure 8, right), support vector machine, decision trees, random forest, and *k*-nearest neighbors only give a single predicted label as the output, while logistic regression and naïve Bayes give multiple predictions, with different weights or probabilities attached to each prediction.

## Unsupervised Learning

In contrast to supervised learning, unsupervised learning attempts to extract meaningful information from unlabeled data. As such, no human input is needed when it comes to data set labeling. This is a significant advantage over supervised learning because of the high labor and time costs of annotating data sets. In this section, five unsupervised learning algorithms across three categories will be discussed. The categories include dimensionality reduction, clustering, and density estimation. Dimensionality reduction algorithms aim to reduce the number of features needed to distinguish between different points in the data set. Clustering algorithms attempt to group data points together that are similar in their features. Density estimation algorithms are designed to approximate the distribution from which the data set is sampled.^77^

### Principal Component Analysis (PCA)

Principal component analysis (PCA) belongs to the family of dimensionality reduction algorithms. PCA is an extremely common method for ascertaining the general trend in your data set, while making it easier to analyze by reducing the number of dimensions.^78^ It essentially creates a working summary of your data with dimensionality suitable for standard algorithms. It requires the use of continuous variables and works best with higher dimensional data sets. Additionally, because it seeks to find overarching patterns in your data, it will be more accurate with larger data sets.

**Figure 9:**
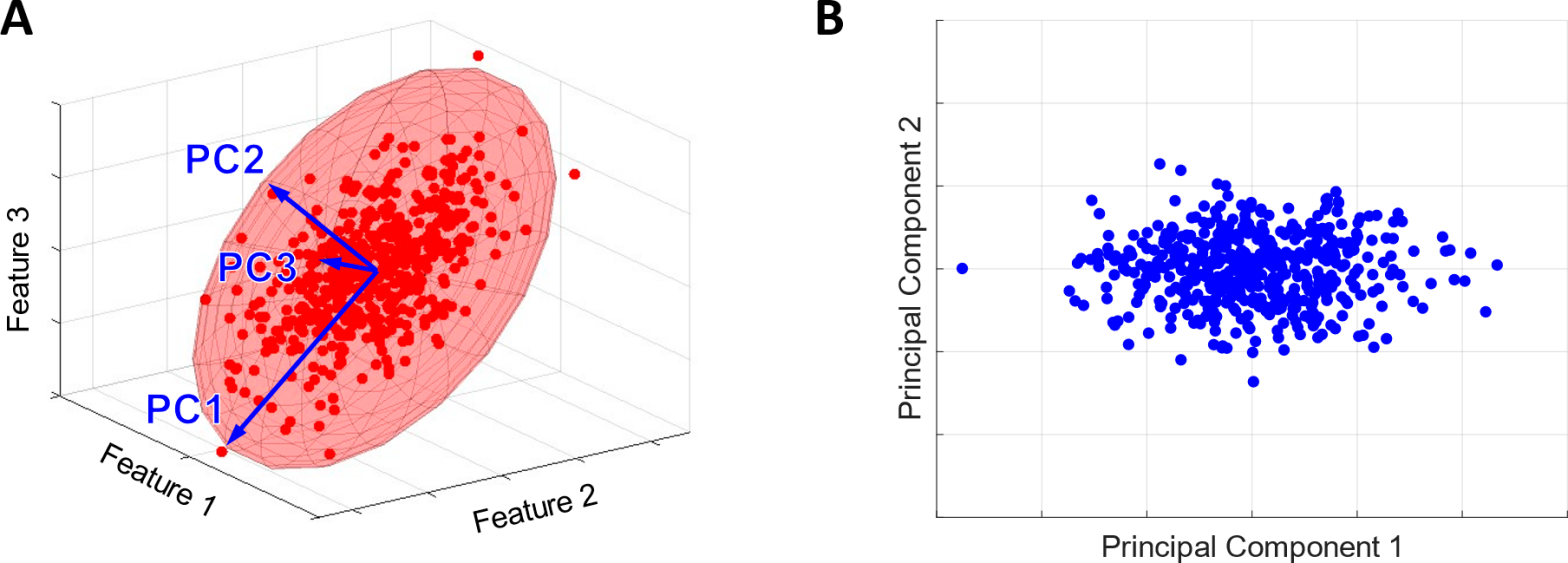
Visualization of the principal component analysis (PCA) algorithm. (A) PCA attempts to fit an ellipsoid over a given data set. Here PCA is applied to a randomly generated 3D data set (red dots), and the fitted ellipsoid is visualized (pale red ellipsoid). The principal components generated by PCA are represented by the direction of the axes, in descending order of their lengths. They are represented as blue arrows, and labeled as PC1, PC2, and PC3 according to their lengths. (B) By only keeping the first few PCs and discarding the rest, a lower-dimensional representation can be obtained without significant loss in the variance represented with the original data. Here, PC1 and PC2 are picked, and the dimensionality of the data set is reduced from 3 to 2.

PCA attempts to fit an ellipsoid (a shape generalized from a 2D ellipse to higher dimensions) to the input data set such that as many input data points as possible are enclosed while keeping the volume low (Figure 9A, red ellipsoid). Then it extracts the direction and length of its axes as its output (Figure 9A, blue arrows). The fitted ellipsoid will have the same number of dimensions as the number of features, and is constructed by calculating the direction of the axes of the ellipsoid. The directions of the axes are called principal components (PCs), and they are numbered according to the length of the axes; PC1 corresponds to the longest axis, PC2 corresponds to the second longest axis, and so on (Figure 9A, blue arrows). In addition, the order of the PCs indicates the quantity of information they carry in terms of how different the data points are from each other. In other words, the features in the direction of PC1 contain the most amount of variance of the data set, and those in the direction of PC2 contain the second most amount of variance, and so on. Even though the number of PCs is the same as the number of features, PCs corresponding to the shortest axes can be discarded with minimal impact on the representation of the input data set since they contribute the least to the overall shape of the ellipsoid (Figure 9B). This is how PCA is utilized for dimensionality reduction. For each PC, a contribution score can be calculated that quantifies the amount of information that it captures. The contribution scores can aid in the decision of the best number of PCs to keep.

PCA originates from mathematical analysis in linear algebra and has found a wide variety of applications in many fields of study including signal processing,^79^ mechanical engineering,^80^ meteorological science,^81^ structural dynamics,^82^ and ML.^83^ It provides a mathematically rigorous and interpretable way of condensing a set of features into a smaller set without losing much information. In ML, PCA is commonly used in conjunction with other algorithms to filter out features that do not contribute significantly to distinguishing the data points, thus reducing the computational load for the downstream algorithm.^83^ However, since PCA relies on linear algebra techniques, the PCs generated by PCA are always linear combinations of some or all of the input features. As a result, PCA may not be suitable for data sets that are highly nonlinear. Additionally, PCA can be easily biased by outliers. Outliers, by definition, lie far away from the majority of the data set. This creates highly elongated axes when PCA attempts to fit an ellipsoid, resulting in PCs favoring outliers over well-behaving data points. Thus, it is important to verify that a given data set is suitable for PCA before applying the algorithm.

PCA has been used for a variety of applications in the field of small molecule design, and a number of examples can be found across a wide range of fields. It has assisted other methods in some notable examples such as its use in identifying the role of small molecule serum metabolites on dengue disease severity,^84^ discovering molecules capable of activating Glucose-6-Phosphate Dehydrogenase,^85^ and for elucidating the various pharmacological properties of essential oils made from *P. senacia* and *S. coriaceum*.^86^ These few examples can hardly begin to highlight the versatility and widespread applicability of PCAs for biological applications, and they should be considered in any cases that could benefit from a reduction in dimensionality.

A more specific example in which PCA was utilized for dimensionality reduction can be seen in the research done by Li et al.^87^ In their work, the authors aimed to generate a model for predicting drug–target interactions using the structural information of both the drug molecules and the target proteins. They devised a novel fingerprinting method to represent drug structures, which includes information about the existence of functional groups and fragments. For protein sequences, they utilized the position-specific scoring matrix method to retain the evolutionary information on the sequences. Before training the ML model, the authors applied PCA to the input features to reduce the computational load and the noise in the data set. The authors then tested their model on multiple existing data sets including Enzyme, GPCR, Ion Channel, and Nuclear Receptor. In all tests, their model achieved high performance through multiple types of quantification including precision, accuracy, and sensitivity.

For mathematically inclined readers, PCA calculates the eigenvalue and eigenvectors of the covariance matrix of the input features. The PCs correspond to the eigenvectors and are ordered according to their eigenvalues. Another algorithm called singular value decomposition (SVD) can directly calculate the PCs from the input features without the need to generate the covariance matrix, but the details are beyond the scope of this review.

### Independent Component Analysis (ICA)

Independent component analysis (ICA) is another unsupervised learning algorithm that, similar to PCA, attempts to dissect the data set into a few key components. These components can then be trimmed down according to their contribution to the data set, thus lowering the dimensionality. However, instead of focusing on the variance of the data set, ICA assumes that the observations in the data set are linear combinations of multiple independent sources, and attempts to mathematically derive a set of independent components (ICs) that represents the most likely set of sources contributing to the observations.^88^ While PCA simply generates the same number of PCs as the dimensionality of the data set, the number of ICs generated by ICA is determined by user input. ICA can be applied to the same data set multiple times with varying numbers of ICs, and the results can be manually inspected to pick the best performing version.

**Figure 10:**
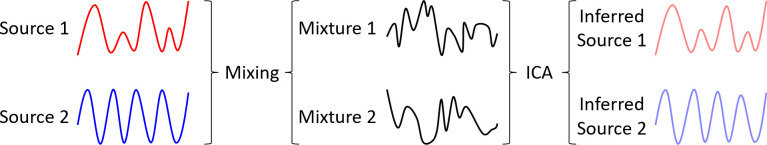
Illustration of the independent component analysis (ICA) algorithm. The task ICA is designed to handle is to infer independent source signals from linear mixtures. The source signals (left, red and blue) are mixed to produce mixture signals (middle, black), and the exact mixing is unknown to ICA. By assuming the source signals are non-Gaussian and attempting to maximize non-Gaussianity, ICA is able to infer the source signals (right, pale red and blue).

One of the classic applications of ICA is the “cocktail party problem”, where the goal is to tease out the voice of a specific person among the mixture of everyone’s voice at a cocktail party. In this case, each person’s voice is an independent source signal, and audio tracks recorded from different parts of the room are the observations or the mixture signals. As a simplified example, suppose there are two people talking in the room providing the source signals (Figure 10, left, red and blue lines), and two recordings from two different corners of the room providing the mixture signals (Figure 10, middle, black lines). Let *s*_1_ and *s*_2_ denote the two source signals and *m*_1_ and *m*_2_ denote the two mixture signals. Assuming the mixtures are linear combinations of the source signals, we have
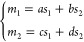


Here, *a*, *b*, *c*, and *d* can be any real numbers and represents the ratio at which the two source signals are mixed to form the mixture signals. With some algebraic manipulations, we can calculate *s*_1_ and *s*_2_ with *m*_1_ and *m*_2_ as follows:
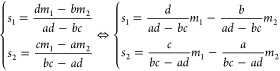


This would be a simple solution to the problem at hand, but we do not have the values of *a*, *b*, *c*, or *d* and thus this solution cannot help us directly. However, this is where ICA comes into play. Let us redefine a few variables in the equations as follows:
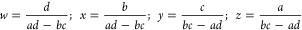


Then the previous equations can be simplified as
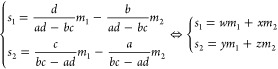


Because we do not have the true values of *w*, *x*, *y*, and *z*, the goal of ICA is to provide the best estimates of these numbers that lead to the best approximations of the source signals. To put it in mathematical terms, let *ŵ*, *x̂*, *ŷ*, and *ẑ* denote the estimated values of *w*, *x*, *y*, and *z*, and *ŝ*_1_ and *ŝ*_2_ denote the estimated source signals. Then the estimated source signal can be calculated as
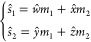


With the goal defined, there is still one key problem: how can one determine whether an estimated source signal is good without knowing the ground truth? The solution lies in a key assumption of ICA: the source signals are non-Gaussian. Non-Gaussian signals are those whose distribution does not follow a Gaussian or a normal distribution. In the case of the cocktail party problem, the voice of an individual is highly unlikely to follow a Gaussian distribution since the pitch of human voices shifts to distinctive ranges in different scenarios (high pitch for questions and exclamations, low pitch for mumbling, medium pitch for explanations). Thus, the immediate metric for evaluating the estimated source signals is to measure their non-Gaussianity. Two notable algorithms that make use of this metric are the projection pursuit algorithm and the FastICA algorithm, both of which attempt to maximize the non-Gaussianity of *ŝ*_1_ and *ŝ*_2_ by tuning the numbers *ŵ*, *x̂*, *ŷ*, and *ẑ*.

An example that utilizes ICA in a domain that is applicable to small molecule discovery can be found in the 2011 study by Debrus et al.^89^ The authors were able to use ICA to separate co-occurring peaks from high-performance liquid chromatography (HPLC) results. This enabled them to screen and separate 19 antimalarial compounds. They provided the gradient time, temperature, and pH as parameters for the model and found that using these features it was able to successfully identify the separate compounds. This approach could easily be applied to small molecule discovery as a means to reduce the guesswork in identification assays.

For the mathematically inclined readers, the details of the derivation of ICA, as well as the projection pursuit and FastICA algorithms, can be found in the review paper written by Alaa Tharwat.^88^

### *k*-Means Clustering

*k*-Means clustering is a clustering algorithm. It is a commonly used method for inferring information from unlabeled data sets, as its primary purpose is to group similar data together and derive overall trends by creating representative clusters.^90^ It can be used only on quantitative and continuous data. While it is possible to use this method for larger data sets, its performance is significantly better on smaller data sets, as various adaptations will be necessary to allow it to scale up to larger data sets due to computational complexities.

**Figure 11:**
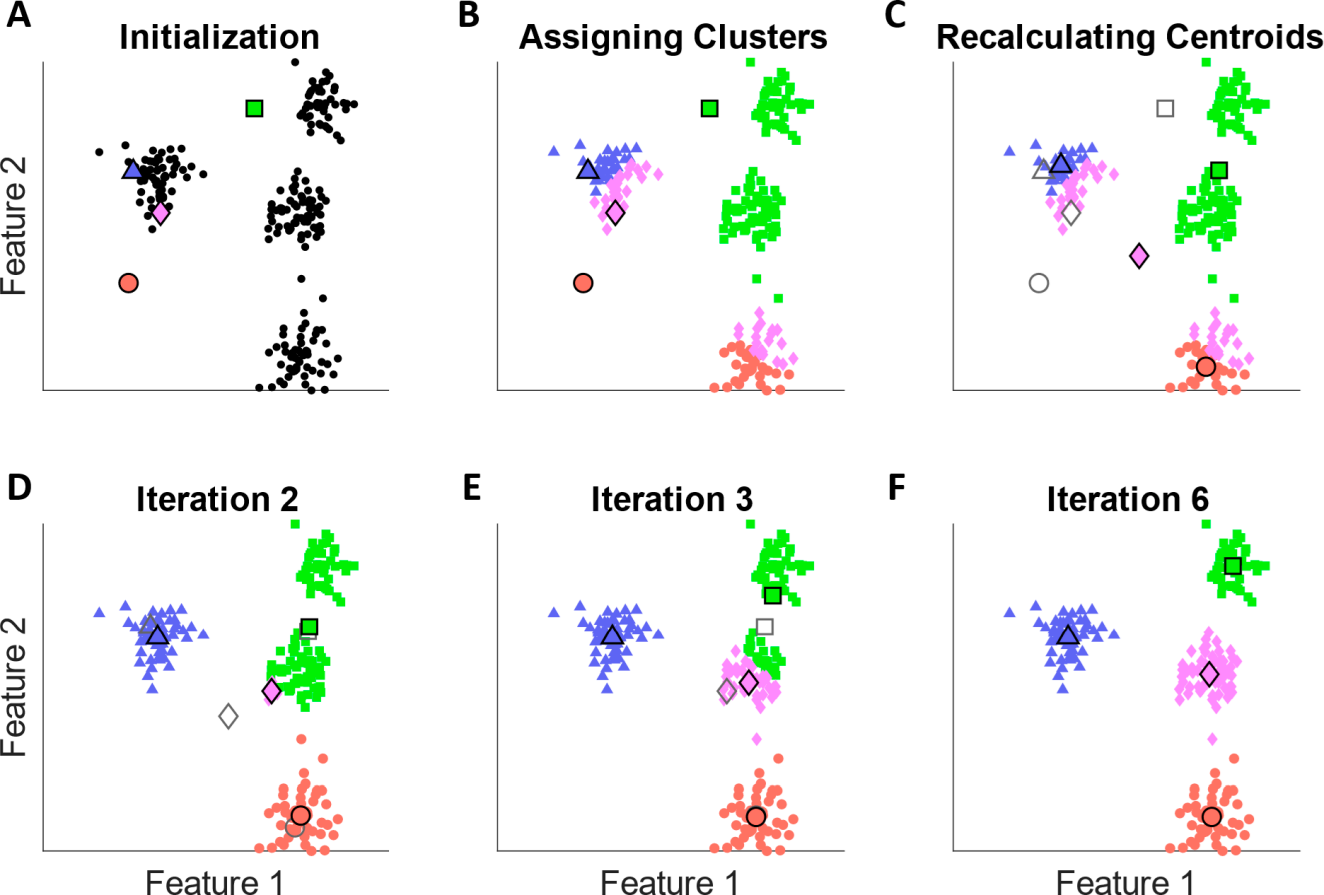
Visualization of the *k*-means clustering algorithm. (A) To initialize the algorithm, the number of clusters are determined (4 in this example) and the centroids of the clusters are randomly generated (colored triangle, diamond, circle, and square). Data points are represented as black dots. (B) The first step of *k*-means clustering is to assign each data point to a cluster. This is done by iterating through all data points, calculating their distances to all cluster centroids, and assigning them to the cluster whose centroid is the closest to them. Here, the cluster assignment is visualized with the color and shape of each data point to match its assigned cluster. (C) The second step is to recalculate the centroid of each cluster. This is done by simply averaging the coordinates of all data points assigned to a cluster. The new centroids are visualized as large colored shapes with black outlines, while the initial centroids are rendered as empty gray outlines. (D, E) The process detailed in panels (B) and (C) are repeated, and the resulting clusters and centroids are visualized in (D) for iteration 2 and (E) for iteration 3. (F) After 6 iterations, the centroids converge, and the algorithm finishes.

The goal of *k*-means clustering is to segment the input data set into *k* groups (called clusters) according to their features, where *k* is a number specified by the user. The algorithm achieves this by finding the center of each cluster, called the centroid, through an iterative process. To initialize the algorithm, *k* random points are chosen as the centroids of the clusters in the feature space (Figure 11A, colored shapes). During each iteration, for each given data point in the input data set, the distances from itself to all the *k* centroids are calculated, and the point is assigned to the cluster whose centroid is the closest to it (Figure 11B). After all data points have been assigned to a cluster, the centroid of each cluster is recalculated by averaging the features of all data points within the cluster (Figure 11C). Then the next iteration starts by reassigning each training data point to the nearest cluster and so on (Figure 11D and E). The process ends if the difference in the centroid positions between consecutive iterations reduces to zero or falls below a preset threshold (Figure 11F). The *k*-means clustering algorithm is guaranteed to reach a stable solution, but the solution is dependent on the initial randomized position of the centroids.^91^ To circumvent this problem, multiple rounds of *k*-means clustering can be performed, and the best performing result can be chosen by picking the clustering that minimizes distances between points in the same cluster and maximizes distances between points from different clusters. Additionally, similar to the *k*-nearest neighbor algorithm, the performance of *k*-means clustering is heavily dependent on the value of *k* that is chosen. With a low *k* value, points far from each other may be grouped into the same cluster; with a high *k* value, one natural cluster of points may be further split apart. One way to narrow the choice of *k* is to train multiple models with different values of *k* and to choose the smallest one with reasonable performance.

An example of *k*-means clustering is found in the 2019 conference paper by Syarofina et al., in which the authors utilize *k*-means clustering, along with a variety of clustering evaluators, to create a model capable of discovering molecules that inhibit dipeptidyl peptidase-4 (DPP-IV).^92^ DPP-IV is a significant target for the treatment of type 2 diabetes mellitus, and the discovery of potential inhibitors for DPP-IV is an important drug development goal. The model discussed here was trained on a set of 100 known DPP-IV inhibitors prior to subsequent cluster evaluation. They were able to identify key molecular properties that can be used to reduce the number of assays necessary for high-throughput screenings in the future.

### Hierarchical Clustering

Hierarchical clustering is another member of the various clustering unsupervised learning algorithms. This clustering method groups similar data points together to understand the overall trend of the data set.^93^ It can be used with either quantitative or qualitative data, but each data set must be limited to one data type and cannot be mixed. Additionally, this method is only applicable to small data sets and should not be used for larger data sets.

**Figure 12:**
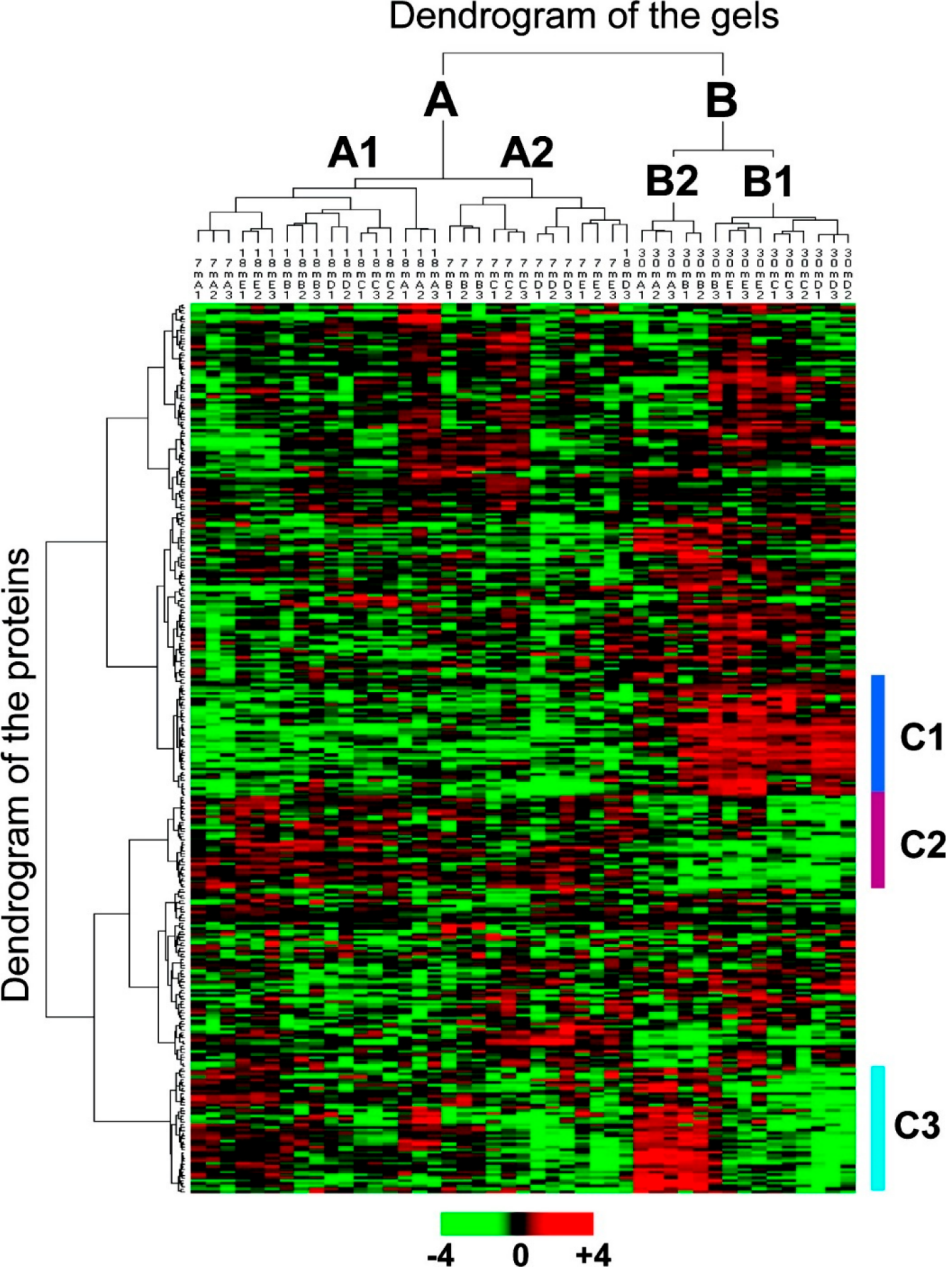
An example of a two-dimensional hierarchical clustering analysis. The data set clustered is a proteomic data pertaining to rat age-related sarcopenia, obtained through 2-D PAGE gels and measured in triplicate. Rows represent proteins, and columns represent gels. Each cell represents the log-ratio transformed amount of protein according to the color bar at the bottom. The dendrogram to the left represents the clustering of the proteins, while the one on the top represents the clustering of the gels. The markers to the right (C1, C2, C3) denote three clusters of proteins that showed similar behaviors across all gels. Reproduced from ref (95). Copyright 2007 American Chemical Society.

Unlike *k*-means clustering, hierarchical clustering does not require the user to specify the number of clusters. Instead, it produces a full binary tree of the input data set, where each split in the tree results in two downstream clusters. The tree ends with every data point in its own cluster, but the branches can be “shaved” to obtain clusters of larger sizes. There are two strategies to carry out hierarchical clustering: to start from one single cluster and keep splitting the cluster into smaller segments (top-down) or to start by treating each data point as its own cluster and gradually merge small clusters into bigger ones (bottom-up). In both strategies, the similarity between data points is the criterion for splitting large clusters and merging small clusters. Different similarity metrics in the form of kernel functions can be employed depending on the types of the input data set.^94^ The resulting tree is commonly visualized as a dendrogram, where the length of the branches is inversely proportional to the similarity between the two split clusters. An example of hierarchical clustering is shown in Figure 12, where the protein concentrations related to rat age-related sarcopenia obtained from 2D PAGE gels is clustered.^95^ The dendrogram on the top shows the clustering of different gels, while the one on the left shows the clustering of different proteins. The protein level of each cell is represented with different colors, according to the color bar at the bottom. Labels C1, C2, and C3 to the right of the figure indicate three groups of proteins that behaved similarly across different gels. Hierarchical clustering has found its uses in many biological contexts including the derivation of phylogenetic relationships between species, the discerning of gene expression patterns from microarray data, and so on. This method is generally useful if the input data set is expected to have a hierarchical structure.

An example of hierarchical clustering is found in the 2022 paper by Teles et al., in which they used hierarchical clustering to build a model to screen for oxazole and oxadiazole derivatives.^96^ These are compounds that are capable of fighting *L. infantum*, the causative agent for the tropical disease Leishmaniasis. They clustered their data based upon structural and conformational features and were able to identify features that can be used for future identification of antileishmanial compounds. Additionally, their model was able to predict the IC_50_ values of potential compounds accurately compared to the experimental results.

### Expectation-Maximization (EM) Algorithm

The final algorithm in this section is the expectation maximization (EM) algorithm. This method works by determining the probability that a given data point belongs to a particular cluster of data points. It can be used with continuous quantitative data and is best suited for smaller data sets. It can be applied to larger data sets as well but would require certain adjustments to make it computationally viable.^97^

This algorithm has found use in the naïve Bayes supervised learning algorithm, but, when taken out of that context, it simply aims to fine-tune the parameters of a probability distribution to best fit a set of observed data and thus belongs to the class of density estimation algorithms.^98^ For EM to work, a probability distribution function with a number of parameters must be assumed. The most commonly used assumption is the Gaussian mixture model. In this model, the distribution is assumed to have many peaks with the distribution immediately around the peaks following a Gaussian or normal distribution. Here, we will use the Gaussian mixture model as our example. EM is an iterative process, starting with a random guess for all of the parameters in the model, which includes the location and the width of each peak (corresponding to the mean and variance of each single Gaussian distribution). Then, for each data point, an estimation is made according to how far it is to each of the peaks and how wide the peaks are, to discern which of the peaks it most likely belongs to. This is called the expectation step. Using these estimations, the locations and widths of the peaks are modified in order to maximize their coverage of the points assigned to them during the expectation step. This is called the maximization step. Through many iterations of the expectation-maximization steps, the parameters will converge to a locally optimal solution near the initial random guess, and the algorithm finishes when the parameters stop changing or their changes fall below a certain threshold. Like *k*-means clustering, which is also an iterative unsupervised learning algorithm, EM is sensitive to the initialization of the parameters.^99^ Thus, multiple rounds of randomized initial parameters need to be carried out to ensure the robustness of the resulting model. Another key to a successful EM model is the choice of the probability distribution. For Gaussian mixture models, the number of independent Gaussian distributions is important for producing a reliable and interpretable overall distribution. Depending on the structure of the input data set, other distributions may provide a better fit than the Gaussian mixture model.

EM algorithm differs from clustering algorithms in that it provides a probabilistic view of the distribution of the input data set instead of a clear-cut grouping or clustering. This may prove to be crucial for small molecule design in capturing the large variations in chemical and biological assays comprehensively. An example of expectation maximization, and more specifically a Bayesian–Gaussian mixture model, can be seen in the 2022 paper by Wei et al., in which the authors created a web interface that is capable of accepting the input of a small molecule and producing an output of potential targets.^100^ This is accomplished by analyzing binding poses before screening them against potential targets. This interface is publicly available online and could help expedite the search for novel drugs.

### Summary

This section introduced five different unsupervised learning algorithms, and they can be roughly divided into three major categories. The first category is called clustering algorithms and aims to segment a given data set according to how similar the data points are within the feature space. To achieve this, both *k*-means clustering and hierarchical clustering resort to generating a well-defined grouping of the data points. While *k*-means clustering is widely applicable to any data set, hierarchical clustering is preferred when the data set comes from a process that is hierarchical in nature such as the evolution of species, functional grouping of proteins, and mutation of genes. The second category is called density estimation, and the expectation-maximization (EM) algorithm belongs to this category. In contrast to clustering algorithms, instead of generating a grouping of the data set, it takes a probabilistic approach and attempts to find a distribution that best fits the data set. Thus, EM is capable of providing a confidence score on whether two points are within the same category instead of a simple yes or no answer. Principal component analysis (PCA) and independent component analysis (ICA) belong to the final category of algorithms, dimensionality reduction algorithms, that aims to reduce the complexity of data sets. They are widely applied to situations where the features in a data set are too numerous to be studied efficiently. They have also been commonly used in conjunction with supervised learning algorithms to reduce computational load by reducing the number of features.

## Advanced Methods

In addition to the many algorithms already introduced in this Review, there are many more ML methods with greater complexity and performance that are highly applicable to small molecule designs. The methods that will be introduced in this section are reinforcement learning, semi-supervised learning, artificial neural networks, and boosting algorithms. These were specifically selected due to their versatility and applicability to small molecule discovery.

### Reinforcement Learning

One class of advanced methods that is particularly notable is reinforcement learning. Reinforcement learning adopts a completely different approach to both supervised and unsupervised learning methods. Instead of predicting the label using a given set of features, it aims to explore an environment by iteratively navigating through it. Reinforcement learning algorithms consist of two parts: one that explores the environment, called the actor, and one that evaluates the actor’s actions, called the critic. For example, suppose we would like to develop a small molecule compound from a known scaffold to bind to a protein. In this case, the environment includes all compounds that can be generated from the scaffold, and the actor would attempt to modify the scaffold by adding or removing atoms. Then, after each round of modification by the actor, the critic would simulate the binding between the new compound and the protein to assign a score according to how strongly the two bind to each other. In the next round, the actor will consider the score from previous rounds and bias its decision on what modification to introduce toward those more similar to those that achieved higher scores. Reinforcement learning has been applied to a wide variety of chemical and biological problems, including omics,^101^ medical imaging,^102^ brain–machine interfaces,^103^ and small chemical compound designs.^104,105^ With recent improvement of simulation capabilities on molecular interactions, due to the development of GPUs and supercomputing centers, reinforcement learning has seen a surge in popularity when it comes to small molecule designs.^106^

In a recent research endeavor conducted by Gottipati et al.,^105^ the authors utilized reinforcement learning to incorporate not only criteria for biological activities but also their synthetical accessibility to make sure the compounds suggested by the algorithm are possible to synthesize chemically. The performance of the new algorithm, the Policy Gradient for Forward Synthesis (PGFS) algorithm, is not compromised by the consideration of ease of chemical synthesis and is comparable with state-of-the-art reinforcement learning algorithms such as Proximal Policy Optimization (PPO) and Actor-Critic using Kronecker-Factored Trust Region (ACKTR). Finally, the authors verified their algorithm in silico, and it successfully generated easy-to-synthesize compounds that target three different HIV-related biological processes.

### Semi-supervised Learning

Another category of advanced ML paradigm is semi-supervised learning. Semi-supervised learning methods have recently risen in popularity, although many of them are much more complex in structure than the algorithms introduced previously. These algorithms are designed to handle data sets that are partially labeled. The goal is to learn the correlation between features and labels from the labeled data points with the assistance from the feature distribution of the unlabeled data points. This is typically achieved by assuming that the population from which the input data set is sampled is likely to be continuous such that unlabeled data points are likely to share the same label as a labeled point nearby. This allows semi-supervised learning to attain comparable performance to fully supervised learning while requiring significantly less human effort to manually annotate data sets, much like unsupervised learning. Semi-supervised learning has been successfully applied to many aspects of small molecule design including predicting activity from chemical structures,^107^ metabolic analysis,^108^ and drug target prediction.^109^

In a study conducted by Bahi and Batouche,^109^ the authors utilized a semi-supervised learning algorithm to predict new drug-target interactions (DTI) using sparsely labeled data. Due to the large amount of information available on drugs and on protein targets, it is infeasible to experimentally test out each pair of drug–protein interactions. Thus, the available DTI data set is mostly unlabeled, with very few pairs reported in experimental literature. The authors developed an algorithm using a combination of deep artificial neural network and semi-supervised learning, termed DeepSS-DTIs, to predict potential drug-target interactions within the data set hosted on the database DrugBank. DeepSS-DTIs achieved an overall accuracy of 98%, and highly ranked predictions are verified through past experimental literature.

### Artificial Neural Networks

A highly powerful, but complicated, class of machine learning algorithms, called artificial neural networks (ANNs), has risen in popularity due to recent advances in computing power, especially in the form of graphical processing units (GPUs). This method is extremely versatile and capable of both classification and regression. It can utilize supervised, unsupervised, and reinforcement learning frameworks and performs best on large data sets. Additionally, due to the wide variety of neural network frameworks, it is possible to utilize a number of different variable types. ANNs can be largely divided into three types: basic, convolutional (CNN), and recurrent (RNN). This section will focus primarily on basic ANNs, but it will also briefly touch on the variations to the technique listed above.

**Figure 13:**
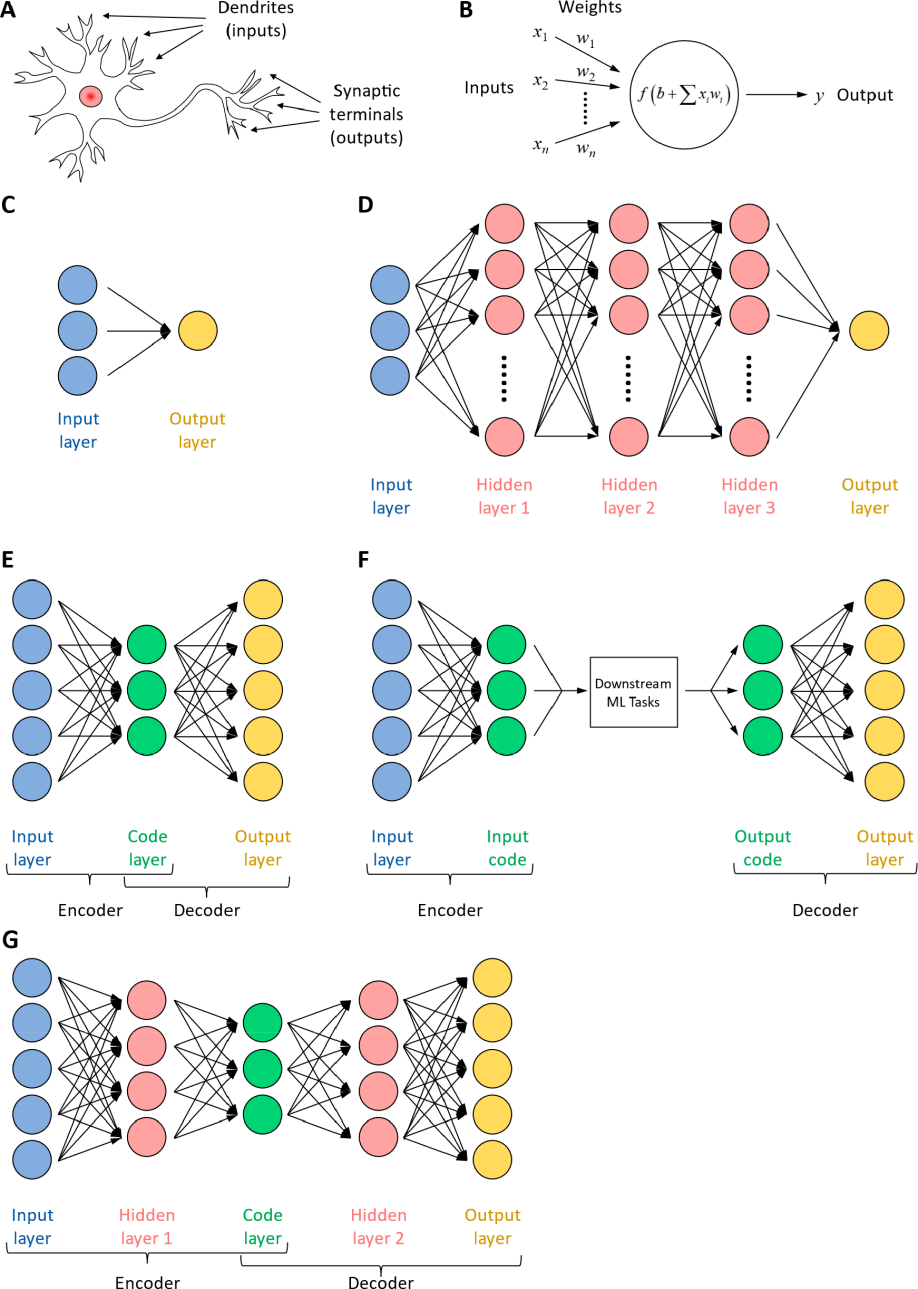
Basics of artificial neural networks (ANNs). (A) A biological neuron receives inputs through its dendrites, processes the inputs, and transmits its output through synaptic terminals. (B) A basic calculation unit, in an ANN. It receives inputs from upstream units, perform a weighted sum of all the inputs plus a bias term, passes the sum through a function called the activation function, and finally outputs the result to downstream units. The calculation units are termed “neurons” due to their similarity to biological neurons. (C) The basic architecture of ANNs consists of an input layer, a hidden layer, and an output layer. Each layer consists of many neurons, and neurons in one layer can only receive inputs from the layer immediately before them. The neurons in the input layer pass the features of the training data set to the hidden layers without calculations. During training, the weights and bias of each neuron are tuned iteratively to improve the accuracy of the output. (D) The architecture of deep learning ANNs. In contrast to basic ANNs, deep learning introduces multiple hidden layers (3 pictured) in between the input layer and the output layer. The training process of deep learning ANNs is the same as basic ANNs. Due to the additional hidden layers, deep learning ANNs are more time-consuming to train, but also perform better on complex tasks. (E) The architecture of a basic autoencoder. It consists of the input layer, the code layer, and the output layer. The input layer and the output layer always have the same number of neurons, while the code layer has fewer neurons than the other two layers. The connections between the input layer and the code layer encodes the input into a low-dimensional representation into the code layer, and the connections between the code layer and the output layer extrapolates the low-dimensional representation into its native form in the output layer. The first two layers are termed the encoder, while the last two layers are termed the decoder. (F) A typical application of an autoencoder. After training the autoencoder, the encoder and the decoder are separated. The encoder is used to generate a low-dimensional representation, and then pass it through a downstream ML task for further processing. After the downstream ML task finishes and generates its output in the low-dimensional representation, the decoder is used to extrapolate back into its native form. (G) The architecture of a deep autoencoder. Instead of a simple 3-layer configuration, additional hidden layers are added within the encoder and the decoder portion of the algorithm (1 layer in the encoder and the decoder illustrated). There is no limit to how many hidden layers can be added, but similar to deep learning, the computational cost increases as the number of hidden layers increases. Panel A created by Jonathan Haas under the CC BY-SA 3.0 license.

ANNs, as the name suggests, imitate the architecture of biological brains and weave a large quantity of simple calculation units called neurons into a web of high complexity. The neurons in ANNs are functionally similar to biological neurons.^110^ In a biological brain, a neuron receives signals from neighbors through dendrites. These signals are processed as input, and the result is sent as output to other neurons at its synaptic terminals (Figure 13A). Similarly, a neuron in an ANN accepts the output of other neurons as its input, performs a weighted sum of the various inputs with an optional bias term (*b* + Σ*x*_*i*_*w*_*i*_, *b*: bias, *x*_*i*_: *i*th input, *w*_*i*_: weight of the *i*th input, Figure 13B), and then passes the sum through a function called the activation function (*f*, Figure 13B). The result is the output of the neuron (*y*, Figure 13B), which is passed on to other neurons in the network. The activation function is useful for limiting the range of the output to avoid negative outputs and/or outputs with extremely large magnitudes.^110^ However, unlike biological brains, where neurons can connect to each other freely, ANNs are based on a layered and hierarchical structure, where neurons on one layer can only pass their outputs to the next layer. The simplest ANN architecture can be seen in the form of the single-layer perceptron, which consists of an input layer and an output layer (Figure 13C). This minimal structure is referred to as a shallow neural network.^111^ The number of neurons in the input layer and the output layer are dependent on the data set that is to be learned. The reduced nature of this structure is beneficial in that it takes less time to train but has the downside of only being capable of learning functions that are linear in nature.

An example of an ANN, and specifically a shallow neural network, can be seen in the 2019 work of Karim et al.^112^ Their study used a combination of a decision tree and a shallow neural network to predict the toxicity of a number of chemicals. This was accomplished by using the decision tree to narrow down the features that are most critical for predicting chemical toxicity, and the shallow neural network to apply the features toward predicting the toxicity of a given chemical. Their shallow neural network consisted of only one hidden layer and ten neurons. This study highlighted the usefulness of shallow neural networks, along with simpler ML designs in general, due to the ability to train the model in a tenth of the time needed to train a more complex deep neural network, with both obtaining comparable results. That being said, this study does not directly compare shallow networks with deep networks as a typical deep neural network is able to derive its own features, while this study used a decision tree to derive the features for the shallow neural network. This separation between the two processes worked well in this case but broke down when the authors attempted to alter the size of the data set.

The next step in architectural complexity is the multilayer perceptron, which can consist of one or more hidden layers. When it contains three or more hidden layers, it can be considered a simple form of deep learning. Deep learning is conceptually quite similar to what has just been described as it is derived from the same idea of using a seemingly biological model of neuronal connection to facilitate an exchange of information between layers of artificial neurons. But deep learning takes that concept one step further by increasing the number of layers of neurons, which, in turn, increases the amount of complexity that the model is capable of. These additional layers are hidden and are responsible for processing the data between the initial and final layer. Returning to the multilayer perceptron, this model is simply a single layer perceptron, but with additional hidden layers added. The number of neurons in the hidden layers is flexible, and these layers enable greater computational complexity. As a result, a multilayer perceptron, in contrast to a single layer perceptron, is also capable of learning functions that are nonlinear in nature. A downside for deep learning has previously been mentioned in that it takes much longer to train the model. However, once that model has been prepared, it can be used for a wide range of similar problems through the utilization of a method known as transfer learning.

Additionally, deep learning models are uniquely suitable for implementing transfer learning.^113−115^ Transfer learning proposes that a machine learning algorithm trained for one task can be partially utilized on a different but similar task to reduce the computational time. With the layered structure of deep learning models, transfer learning can be implemented by simply replacing the last hidden layer of a pre-trained network with a set of new neurons and performing a quick training only on these neurons. This process is called fine-tuning. During fine-tuning, the amount of training data required is also significantly reduced compared to training a full deep learning model. For example, if a deep learning model has already been trained to distinguish between pictures of dogs and cats, then that same network can be adapted to distinguish between pictures of wolves and cheetahs by retraining the last hidden layer with a relatively small amount of training data.^114^

The most recent demonstration of deep learning is the success of AlphaFold, reported in literature in 2021.^116^ The algorithm first processes the input of raw amino acid sequences through repeated layers of a novel deep learning architecture, termed the Evoformer, to derive spatial and evolutionary information. This information is then passed through a structure prediction model that iteratively refines the rotation and translation of each residue of the protein. The performance of the AlphaFold algorithm was demonstrated during the Critical Assessment of Structure Prediction round 14 (CASP14), a biannually community wide experiment to determine and advance the state of the art in modeling protein structures. AlphaFold outperformed all other participants in CASP14 by a significant margin. The median error rates of AlphaFold predictions are about 60–65% lower than those of the next best performing method.^116^

Following from the multilayer perceptron, the next relevant step in ANN models is autoencoder. Autoencoders are designed to learn encodings of a given set of data. An autoencoder consists of two parts: the encoder and the decoder (Figure 13E). The encoder learns to map the features in the input data from a high dimensional space to a low dimensional space, while the decoder learns to extrapolate representations in the low dimensional space back into the high dimensional space, where the features reside. After the training has finished, the encoder is used to create reliable low-dimensional representations of the input data, and further ML tasks can be conducted on the encoded representations (Figure 13F). The encoded representations offer accelerated learning for downstream tasks due to the lower dimensionality. Finally, since the output of the downstream ML tasks will be in the format of the encoded representation, the decoder part of the autoencoder can extrapolate the results back into the initial feature space.

While the simplest autoencoder consists of only three layers, the input layer, the code layer, and the output layer (Figure 13E), additional hidden layers can be added to the architecture to form a deep autoencoder (Figure 13G). These hidden layers can assist with improving the accuracy of the low dimensional representation and can be crucial when the input data is highly complex. However, like any deep learning ANNs, the addition of hidden layers always carries computational burdens, and too many hidden layers may render the algorithm intractable.

As a dimensionality reduction algorithm, autoencoders are especially attractive for small molecule design because they inherently provide a method to recover the native representation from the low-dimensional representation via the decoder. In a study conducted by Gómez-Bombarelli et al., the authors trained two autoencoders on the SMILES string of 108,000 molecules from the QM9 data set and those of 250,000 drug-like molecules extracted at random from the ZINC database, respectively.^117^ The autoencoders were able to generate low-dimensional representations of these molecules that accelerated the downstream prediction tasks. Additionally, the properties of the molecules decoded from the predictions were comparable to those predicted by using one-hot encoding of the SMILES string.

Moving on from the more basic models of ANN, there are two additional types that will be only briefly touched upon due to their complexity but warrant mention because of their potential applicability to molecular discovery efforts. These types are the convolutional neural network (CNN) and the recurrent neural network (RNN). CNNs differ from basic ANN in that they were initially designed to process images in a way that mimics how the brain processes visual signals from the eyes. The key distinction between a CNN and a regular ANN is that a CNN makes use of the mathematical operation called convolution. Convolution allows for the individual items in the visual data to be identified and mapped as distinct features. This causes it to be extremely useful for processing visual data, which can also be extrapolated to include molecular structures.

The last type is the recurrent neural network (RNN). RNNs are distinct from both ANN and CNN due to their lack of directional limitations. ANNs and CNNs are both feed-forward neural networks, meaning that data are processed from input to output and never the other way around. RNNs break away from this paradigm by allowing their data to go in multiple directions at once, with the output of downstream layers passing back to upstream layers to be reanalyzed as input, thus further refining the model. RNN is most suitable for use with data that are sequential in nature, and has been used for a wide variety of applications such as in linguistic, musical, and genetic data.^118^ However, it can also be seamlessly applied to problems in the chemical space and has been robustly utilized for de novo molecular generation.

### Boosting Algorithms

Boosting algorithms aim to iteratively train new models of a given supervised learning algorithm by adjusting the weights of the input data points according to whether they were correctly predicted in the past iteration. The final model is a sum of all of the models trained during the process. This idea surfaced around the early 1990s and has seen extensive development ever since.^119^ Boosting algorithms are commonly applied to decision trees but can be used for other models as well.^120^ Because of their interdependence with other models, their variable and data set requirements can be determined from the requirements of the parent model. Two of the most commonly used boosting algorithms are gradient boosting and AdaBoost.

Gradient boosting comes from the concept of gradient descent, the same concept that is used to iteratively find the best parameters for linear regressions. Here, instead of applying it to a single model, gradient descent is applied to the model generated from the previous iteration to guide the construction of the next model.^121^ Gradient boosting is an iterative process. To initialize, a basic model is used to predict the training data set, most commonly a constant function. Then, for each iteration, the error of the model from the previous iteration is calculated, and using gradient descent, a new function is calculated to minimize the error. Then the new function is added to the model from the previous iteration to generate a new model, and the loop continues. The algorithm stops when the prediction error of the model falls below a given threshold. It may not be entirely clear as to in which step the input features are reweighted, but the process of calculating and minimizing the prediction error has a similar effect since data points with higher error are prioritized due to their significant contribution to the total prediction error. Since the formulation of gradient boosting does not specify the type of model, it is applicable to a wide range of supervised learning algorithms.

Another commonly used boosting algorithm is called AdaBoost, which stands for adaptive boosting. AdaBoost is specifically designed for classification tasks, i.e., supervised learning to predict discrete labels, and is particularly well-suited for algorithms that are prone to overfitting.^122^ AdaBoost starts with a basic model generated for a given data set, where each data point has equal weighting. Then for each iteration, AdaBoost increases the weighting of the misclassified data points and decreases the weighting of those successfully predicted by the model. In other words, data points whose labels were not correctly predicted receive an increase in their weighting and vice versa. A new model is then generated using the reweighted data set. This process is repeated for a number of times that is defined by the user, and the final model is the average of all of the previously generated models.

An example of both gradient boosting and AdaBoost can be seen in the 2022 paper by Moinul et al., in which the authors compare a variety of ML methods to identify molecules capable of inhibiting sodium glucose cotransporter 2.^123^ The inhibition of these transporters is important for the potential discovery of antidiabetic drugs. The authors utilized nine models to screen for potential inhibitors, with those using gradient boosting or AdaBoost being among the top performers. Though it was not experimentally validated, the model itself holds promise for reducing the number of assays necessary for future experimental projects.

### Summary

This section introduced four additional methods that provide a more complex glimpse into possible machine learning approaches. The first of these methods was reinforcement learning, which is a category in its own right and involves pitching an actor to explore an environment against a critic that judges how well the actor navigates the environment. The second type of algorithm is the semi-supervised learning, which is capable of utilizing partially labeled data sets for label prediction. Another method introduced was ANN, which attempts to conceptually utilize the unique neural networks of biological systems to create an ML model. This model is capable of facilitating an interplay between layers of artificial neurons to produce versatile and highly transferable outputs. The final topic introduced was boosting algorithms, which modify the importance or weight of training data points according to whether they were predicted correctly in the previous repeat. These algorithms provide an accelerated approach for enhancing the performance of repeats of the same algorithm and may prove valuable when a particular algorithm cannot achieve reasonable performance through simple repeats alone.

## Additional Topics

In the previous sections, many ML algorithms were introduced. Each has its own advantages and disadvantages. Some are suitable for predicting categorical labels, while others can generate continuous labels. In this section, we will cover a few overarching topics about ML algorithms as a whole including ensemble methods, the problem of overfitting, and available coding resources.

### Ensemble Methods

The concept of combining multiple ML algorithms came from the simple fact that each ML algorithm has its own advantages and disadvantages, and the combination of many may provide a more comprehensive solution to the task at hand. By utilizing many different algorithms for the same problem, the modeling and prediction results can potentially be improved. However, the amount of computation required scales quickly with the number of algorithms combined; therefore, it is not a widely utilized modality compared to the use of singular algorithms. On the other hand, its utility has been demonstrated in the 2022 study by Grimberg et al., in which they combined lasso regression, a decision tree, and a convolutional neural network to identify small molecules capable of targeting the RNA hairpin in the ribosomal peptidyl transferase region of *M. tuberculosis*.^124^ The activity of a number of these predicted molecules was confirmed experimentally, and 4 out of 10 of those synthesized resulted in the inhibition of protein translation in the bacterium. This success rate is a significant improvement upon traditional high-throughput screening methods.

An additional example of ensemble methods can be seen in the 2021 paper by Wani and Roy, in which the authors worked to create ML models to predict small molecules with the potential to fight tuberculosis.^125^ After creating a wide variety of models, they settled on a three-pronged approach utilizing an Adaboost decision tree, a random forest classifier, and a *k*-NN model. These three models were able to create consensus predictions that were far more reliable than any single model on its own and are believed to have the potential to increase the success rate for later experimental screenings.

### Overfitting

For supervised learning and ensemble learning that incorporates supervised learning, overfitting is a common problem. Overfitting occurs when the model fits not only to the underlying correlation but also the noise within the training data. It is usually characterized by impressive training accuracy but poor testing accuracy on data not used during training. A common method of reducing overfitting is called *k*-fold cross validation.^126^ In *k*-fold cross validation, the input data set is, as usual, split into a training data set and a testing data set. Then the training data set is further split into *k* subsets, each with the same number of data points. One of the *k* subsets is held out, and the rest of the (*k* – 1) subsets are used to train a model, while the held-out subset is subsequently used to validate the model. This process is repeated until *k* unique models have been trained, corresponding to each of the *k* subsets being held out. Finally, the *k* models are averaged in a weighted fashion according to their validation accuracy. The averaged model is then validated again using the initial testing data set. The *k*-fold cross validation method is capable of reducing the risk of overfitting, although at the cost of increased computational loads.

Another common source of overfitting is the number of parameters in the model. While a model with more parameters can accommodate more complex data sets, having too many parameters will result in the model learning from random fluctuations in the data set, especially when the number of parameters is larger than the number of data points in the training data. This form of overfitting can be mitigated through a process called regularization.^127^ The goal of regularization is to penalize the model for using too many nonzero parameters. To achieve this effect, a penalty is added to encourage the algorithm to minimize the magnitude of its parameters. This penalty is usually proportional to the sum of the absolute values of all parameters in the model or any other methods of quantifying the total magnitude of the parameters. By adding this penalty, the algorithm will see a decrease in its performance score when any of the model parameters deviate significantly away from zero, thus encouraging it to shrink their magnitude. Regularization in ML can also be seen as an application of Occam’s razor. With the increase in the number of parameters in a model, more assumptions are made about how the data set is structured. Since every assumption has a chance to be wrong, a complex model has a greater chance of failing due to incorrect assumptions compared to a simple model. Thus, if a simple model and a complex model have comparable performance on a task, then the simpler model is preferred.

### Coding Resources

With all of the ML algorithms covered, one may wonder how to implement these algorithms without an extensive mathematical and programming background. In this section, we will cover a variety of existing programming tools and libraries that will make it a much simpler task to apply the algorithms to the problem at hand.

When it comes to machine learning, the most popular programming language is Python. Python is designed to be easily readable, with most of its keywords in English instead of using punctuation marks. It uses indentations to delimit blocks of code, and no semicolons are needed after statements. Many libraries have been written for Python to take care of many basic tasks in research including and not limited to data formatting, manipulation, visualization, and so on. A few of the most commonly used Python libraries are NumPy (support for large arrays and matrices and high-level mathematical functions), pandas (general data manipulation and analysis), and Matplotlib (plotting and visualization library). For machine learning algorithms, there are also quite a few open source and free libraries with implementations of many of the algorithms mentioned in this review. The scikit-learn library is one such library, which features implementations of many algorithms including linear regression, *k*-nearest neighbors, support vector machines, random forests, principal component analysis, and *k*-means clustering, among many others. It also contains useful tools to help with preprocessing, feature extraction, and normalization of your data. When it comes to artificial neural networks and deep learning, libraries such as PyTorch and TensorFlow provide ready-to-use scaffolds for assembling a neural network suitable for your specific needs. Layers of neurons can be easily generated and linked to each other with simple built-in functions, and the libraries also support advanced architectures including convolutional and recurring neural networks. With such a wide choice of libraries, it may seem rather overwhelming to get all of the software packages installed and configured. However, the free Python distribution called Anaconda has all of the aforementioned libraries preinstalled. Anaconda is also available for Windows, MacOS, and Linux, making it a versatile platform for machine learning endeavors regardless of your operating system. Anaconda also provides a user-friendly graphical interface called the Jupyter Notebook, where short sections of code can be tested and debugged immediately instead of having to finish the full Python script. If a standalone installation is too complicated, then Google also provides a browser-based online solution for Python coding called Google Colab. The interface of Google Colab is highly similar to that of Jupyter Notebook, but all of your code will be executed on cloud computing resources hosted by Google. It enables online collaboration, and you can directly access files on Google Drive. One caveat for Google Colab is that there are limitations on the amount of computing power you are allocated, so if your ML algorithm is computationally heavy, a standalone installation of Python (such as Anaconda) may be necessary.

While Python is the most popular platform for ML, there are alternatives if Python is not your preferred choice. One popular coding language, MATLAB, provides two toolboxes related to ML: the Statistics and Machine Learning Toolbox and the Deep Learning Toolbox. The Statistics and Machine Learning Toolbox provides implementations of simple ML algorithms such as *k*-means clustering, hierarchical clustering, SVM, linear regression, PCA, and shallow ANN. The Deep Learning Toolbox contains tools to design deep neural networks using a graphical user interface, preprocess your raw data, create comparisons with built-in pre-trained models, and so on. Wolfram Mathematica also provides a suite of built-in functions to help with ML tasks. Implemented ML algorithms in Mathematica include, but are not limited to, decision tree, logistic regression, random forest, SVM, *k*-means clustering, autoencoder, ANN, CNN, RNN, and so on.

## Conclusion

The field of small molecule design has seen a vast amount of development in the past century and has evolved from relying on natural extracts for compound discovery to high-throughput synthesis of large quantities of molecules from existing scaffolds. However, as the scale of experimental efforts increases, the amount of time, labor, and cost required for such endeavors will quickly exceed the capabilities of academic research institutions. ML is an excellent method for accelerating the progress of research and reducing the time and labor requirements for small molecule design. Through the application of supervised learning, unsupervised learning, and advanced methods, new information can be inferred from chemical and biological data sets from past literature, and molecular predictions can be produced that are highly likely to result in the desired effects. These predictions may assist in solving many modern biological and chemical problems including, but not limited to, antibiotic resistance,^128^ cancer treatment,^129^ cardiovascular disease,^130^ and catalyst design.^131^ By utilizing the computational power of ML algorithms, the number of candidates to test will be vastly reduced, and the hit rate will be increased, thus greatly alleviating the demands on time and effort. As the field of ML develops, more and more computational methods will be developed to be tailored to the needs of small molecule design and will propel the field of small molecule design to new heights.
